# *Plasmodium falciparum* K13 Mutations Differentially Impact Ozonide Susceptibility and Parasite Fitness *In Vitro*

**DOI:** 10.1128/mBio.00172-17

**Published:** 2017-04-11

**Authors:** Judith Straimer, Nina F. Gnädig, Barbara H. Stokes, Michelle Ehrenberger, Audrey A. Crane, David A. Fidock

**Affiliations:** a Department of Microbiology & Immunology, Columbia University College of Physicians and Surgeons, New York, New York, USA; b Division of Infectious Diseases, Department of Medicine, Columbia University College of Physicians and Surgeons, New York, New York, USA; NIAID/NIH

**Keywords:** K13/Kelch13, *Plasmodium falciparum*, artemisinin, drug resistance, fitness, gene editing, ozonides

## Abstract

The emergence and spread in Southeast Asia of *Plasmodium falciparum* resistance to artemisinin (ART) derivatives, the cornerstone of first-line artemisinin-based combination therapies (ACTs), underscore the urgent need to identify suitable replacement drugs. Discovery and development efforts have identified a series of ozonides with attractive chemical and pharmacological properties that are being touted as suitable replacements. Partial resistance to ART, defined as delayed parasite clearance in malaria patients treated with an ART derivative or an ACT, has been associated with mutations in the *P. falciparum K13* gene. In light of reports showing that ART derivatives and ozonides share similar modes of action, we have investigated whether parasites expressing mutant K13 are cross-resistant to the ozonides OZ439 (artefenomel) and OZ227 (arterolane). This work used a panel of culture-adapted clinical isolates from Cambodia that were genetically edited to express variant forms of K13. Phenotypic analyses employed ring-stage survival assays (ring-stage survival assay from 0 to 3 h [RSA_0–3h_]), whose results have earlier been shown to correlate with parasite clearance rates in patients. Our results document cross-resistance between OZ277 and dihydroartemisinin (DHA), a semisynthetic derivative of ART, in parasites carrying the K13 mutations C580Y, R539T, and I543T. For OZ439, we observed cross-resistance only for parasites that carried the rare K13 I543T mutation, with no evidence of cross-resistance afforded by the prevalent C580Y mutation. Mixed-culture competition experiments with isogenic lines carrying modified *K13* revealed variable growth deficits depending on the K13 mutation and parasite strain and provide a rationale for the broad dissemination of the fitness-neutral K13 C580Y mutation throughout strains currently circulating in Southeast Asia.

## IMPORTANCE

ACTs have helped halve the malaria disease burden in recent years; however, emerging resistance to ART derivatives threatens to reverse this substantial progress. Resistance is driven primarily by mutations in the *P. falciparum K13* gene. These mutations pose a threat to ozonides, touted as promising alternatives to ARTs that share a similar mode of action. We report that DHA was considerably more potent than OZ439 and OZ277 against ART-sensitive asexual blood-stage parasites cultured *in vitro*. We also document that mutant K13 significantly compromised the activity of the registered drug OZ277. In contrast, OZ439 remained effective against most parasite lines expressing mutant K13, with the exception of I543T that merits further monitoring in field-based OZ439 efficacy studies. K13 mutations differed considerably in their impact on parasite growth rates, in a strain-dependent context, with the most prevalent C580Y mutation being fitness neutral in recently culture-adapted strains from Cambodia, the epicenter of emerging ART resistance.

## INTRODUCTION

The substantial decline in malaria cases (22%) and deaths (50%) worldwide since 2000 can be largely attributed to the implementation of artemisinin (ART)-based combination therapies (ACTs), as first-line treatment in countries where malaria is endemic, and to expanded *Anopheles* mosquito vector control measures (1). However, the emergence and rapid spread of artemisinin-resistant strains of *Plasmodium falciparum* in the Greater Mekong subregion (historically the epicenter of antimalarial drug resistance) threaten ongoing efforts to eradicate this leading infectious cause of infant mortality. The situation could turn into a public health calamity if ART resistance manifests on the African continent, home to 90% of the malaria-related cases and deaths (2).

ART (a natural product from the Chinese sweet wormwood *Artemisia annua*) and its clinically employed semisynthetic derivatives, namely, dihydroartemisinin (DHA), artemether, and artesunate, are fast acting and potent antimalarial agents that can reduce the biomass of drug-sensitive asexual blood-stage parasites by up to 10^4^-fold every 48 h (corresponding to one generation of the intraerythrocytic developmental cycle). A major limitation of these compounds is their very short half-life in plasma (<1 h), necessitating the addition of a longer-lasting partner drug to clear the infection (3). Emerging resistance to ART was first documented as prolonged parasite clearance times in *P. falciparum*-infected patients treated with artesunate in western Cambodia (4, 5). As a result, ACT partner drugs encounter larger numbers of parasites in ART-resistant infections, increasing the potential for partner drug resistance. Indeed, resistance to mefloquine and piperaquine has been documented in Cambodia, where it has been associated with high levels of ACT treatment failures, as measured by 28-day follow-up efficacy studies (6–9).

A genetic marker for ART resistance has been mapped to the *P. falciparum K13* (*Kelch13*) locus by means of whole-genome sequencing of *in vitro* ART-pressured parasites as well as ART-resistant or -sensitive Cambodian clinical isolates (10). Multiple mutations have been found throughout K13, often situated within the six-bladed β-propeller domain of this protein (11–14). Gene editing experiments with several K13-propeller mutations, including R539T, I543T, and C580Y, have confirmed their causal role for *in vitro* ART resistance in Southeast Asian field isolates as well as in laboratory-adapted strains (15, 16).

Starting with the epicenter of K13-mediated ART resistance in western Cambodia, mutations have now spread to all neighboring countries and beyond, including Vietnam, Laos, Thailand, China, Myanmar, Bangladesh, and India (12, 17, 18). The most predominant mutation is C580Y, which has reached fixation in parts of Cambodia and near the Thailand-Myanmar border (9, 10, 13, 17, 19, 20). The prevalence of the C580Y mutation across all of Southeast Asia has been reported to be 26.5% to 48.3%, with some regions reporting a prevalence of up to 74% (12, 21, 22). The R539T mutation confers high levels of *in vitro* resistance and has been associated with delayed parasite clearance in patients; however, compared to C580Y, it is less prevalent, with 3.5% of K13 mutations in Cambodia-Vietnam-Lao People’s Democratic Republic (PDR) and 0.3% in Thailand-Myanmar-China (10, 12, 13, 15). Similarly, the I543T mutation has been associated with increased survival *in vitro* and a longer parasite clearance half-life in patients, but its prevalence was recently reported to reach only 2% in Southeast Asia (22). One potential explanation for the relatively high prevalence of the C580Y mutation, despite its lower degree of resistance observed *in vitro*, would be its enhanced fitness relative to other mutant isoforms. One measure of fitness is the rate of parasite asexual blood-stage growth (23), which we quantify herein using isogenic parasite lines that express mutant or wild-type K13.

Thus far, no fully effective alternative first-line treatment is available to replace ACTs, spurring efforts to find new potent antimalarial drugs (24, 25). Ideally, the new molecules should not be affected by the current resistance mechanisms, should be able to achieve full cure with a single-dose regimen, and should not depend on a natural product whose supply levels can vary. ART, which possesses a 1,2,4-trioxane heterocycle core, has inspired the development of structurally diverse fully synthetic peroxide antimalarials, including the first synthetic ozonide OZ277 (arterolane maleate, RBx 11160) and the “next-generation” ozonide OZ439 (artefenomel) (26–28) (see Fig. S1 in the supplemental material). Importantly, both ozonides exhibit longer half-lives in plasma compared to the endoperoxide DHA. The half-life of OZ277 in plasma is only 2- to 3-fold longer than for DHA, with a lower plasma exposure in *P. falciparum*-infected patients compared to healthy volunteers (29, 30). OZ277 is nevertheless available in India in combination with piperaquine (Synriam) (31). In contrast, OZ439 exhibits a plasma half-life of 42 to 62 h and shows no reduced drug exposure in patients with acute malaria (32, 33). This promising candidate for a single-dose oral cure, with a good safety profile and low projected cost of goods, is currently undergoing clinical phase 2 trials in combination with ferroquine (ClinicalTrials.gov NCT02497612). OZ439 shows very promising antimalarial efficacy (against both *P. falciparum* and *Plasmodium vivax*) and excellent absorption, distribution, metabolism, and excretion properties (32).

10.1128/mBio.00172-17.2 FIG S1  Structures of endoperoxide antimalarials. Chemical structures are shown for the lactone artemisinin (ART), its active metabolite and lactol derivative dihydroartemisinin (DHA), and the fully synthetic ozonides OZ277 (arterolane) and OZ439 (artefenomel). Artemisinin derivatives are the core component of current first-line ACTs. Arterolane is registered for use in combination with piperaquine in India as well as in several African countries, while the efficacy of artefenomel monotherapy and combination therapy is currently being assessed in phase II clinical trials. Download FIG S1, PDF file, 0.1 MB. Copyright © 2017 Straimer et al. 2017 Straimer et al. This content is distributed under the terms of the Creative Commons Attribution 4.0 International license.

ART and ozonides contain the same bioactive endoperoxide bridge (Fig. S1) and are thought to be similarly activated via iron-catalyzed reductive scission of the endoperoxide bond, with parasite-digested hemoglobin providing a major source of iron activator (34). One leading model is that this reaction generates free carbon-centered radicals, which in turn alkylate parasite proteins as well as other biomolecules leading to parasite killing (35–37). ART derivatives and ozonides both display very similar activity profiles throughout the parasite life cycle, including activity against all stages of asexual blood-stage development, including early rings (38, 39). Here, we examine whether K13 mutations that confer ART resistance also mediate cross-resistance to OZ277 and OZ439. These studies employ a series of culture-adapted isogenic Southeast Asian parasite lines engineered to express mutant or wild-type K13 (15).

## RESULTS AND DISCUSSION

### DHA is a more potent inhibitor of parasites expressing wild-type K13 *in vitro* compared to OZ439 and OZ277.

**FIG 1:**
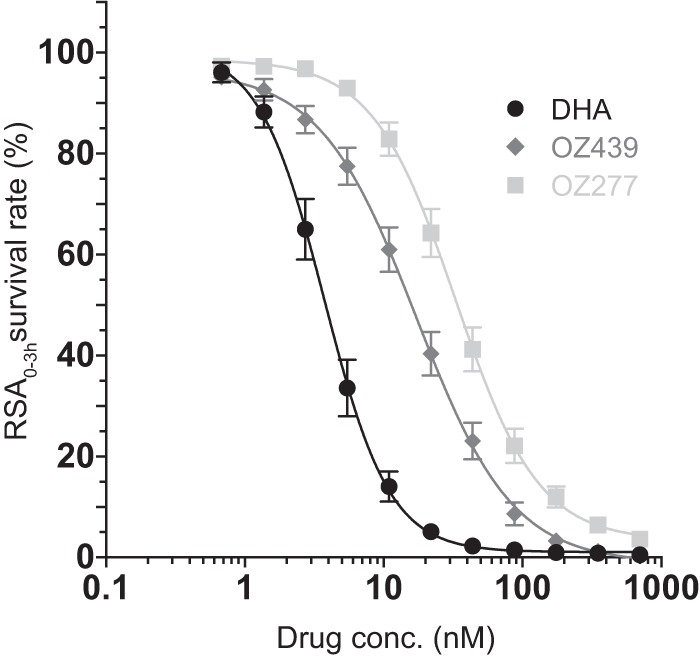
DHA is a more potent inhibitor of parasite survival *in vitro* than OZ439 or OZ277, as defined in the ring-stage survival assay from 0 to 3 h (RSA_0–3h_). Results show the percentage of early ring-stage parasites expressing wild-type K13 (0 to 3 h postinvasion of human erythrocytes) that survive exposure to a 4-h pulse of DHA, OZ439, or OZ277 ranging in concentration from 700 nM to 0.6 nM, as measured by flow cytometry 68 h later. Data show mean ± standard error of the mean (SEM; error bars) percent survival from at least three independent experiments performed in duplicate compared with DMSO vehicle-treated parasites processed in parallel. Data show the combined results for percent survival in four parasite lines harboring the wild-type *K13* allele: CamWT, Cam3.II^rev^, V1/S^ctrl^, and Cam5^rev^. RSA_0–3h_ dose-response curves are shown for DHA, OZ439, and OZ277.

In light of the structural similarities and presumed similar modes of action between ozonides and DHA, we first examined their *in vitro* parasite killing capacities against ART-sensitive parasites (Fig. 1). Using the ring-stage survival assay (ring-stage survival assay from 0 to 3 h [RSA_0–3h_]) that correlates with delayed parasite clearance (40), we assessed the susceptibility of 0- to 3-h ring stages of three recently culture-adapted Cambodian clinical isolates (Cam3.II^rev^, CamWT, and Cam5^rev^ [Cam stands for Cambodian, rev stands for revertant of the mutant allele back to its wild-type sequence, and WT stands for wild type]) as well as an older reference line from Vietnam (V1/S). Each of these *P. falciparum* lines expresses a wild-type *K13* allele (see Table S1 in the supplemental material). The standard RSA_0–3h_ determines the percentage of early ring-stage parasites that survive a single pharmacologically relevant dose of drug, i.e., 700 nM for DHA applied for 6 h. The rate of survival measured *in vitro* has been shown to correlate well with the lengthened parasite clearance half-lives observed in patients infected with ART-resistant malaria (40). With ART-resistant infections, these clearance half-lives (i.e., the time required to reduce the parasite biomass by half) are generally >5 h, in contrast to ART-sensitive infections that show half-lives closer to 2 to 3 h (41).

10.1128/mBio.00172-17.6 TABLE S1  Geographic origin, native *K13* allele, and drug resistance genotypes of *Plasmodium falciparum* clinical isolates and reference lines. Download TABLE S1, PDF file, 0.1 MB. Copyright © 2017 Straimer et al. 2017 Straimer et al. This content is distributed under the terms of the Creative Commons Attribution 4.0 International license.

To obtain a comprehensive evaluation of the *in vitro* potency of OZ439 and OZ277, we modified the assay by exposing early ring-stage ART-sensitive parasites to a gradient of drug concentrations ranging from 700 nM to 0.6 nM for 4 h. Our protocol also employed multiple steps of washing and plate transfers to remove residual drug (see Materials and Methods), as a prior study reported that for OZ277 and more so for OZ439, the single washing step employed in the original RSA_0–3h_ protocol failed to eliminate these ozonides and thus overestimated their activity (39) (a finding that we also observed). From these results, we determined the drug concentration required to inhibit 50% survival, defined as the RSA_0–3h_^50%^. This value was derived following nonlinear curve fitting of log-transformed data combined across our four parasite lines expressing the wild-type *K13* gene, listed above. Results showed that DHA (3.7 nM) was more potent than OZ439 (17.2 nM) and OZ277 (33.1 nM) in its mean 50% inhibition of parasite survival (i.e., RSA_0–3h_^50%^), with the difference in efficacy being the most striking at concentrations below 45 nM (Fig. 1). As an example, only 5.1% of ring-stage ART-sensitive parasites (assayed at 0 to 3 h postinvasion) survived a 4-h pulse of 22 nM DHA, compared to 40.3% and 64.3% that survived a pulse with the same concentration of OZ439 and OZ277, respectively (Fig. 1).

The reduced parasite killing activities of OZ439 and OZ277 agree with the longer parasite clearance half-lives observed in ozonide-treated malaria patients (4 to 6 h and ~9 h for OZ439 and OZ277, respectively) compared to DHA (2 to 3 h) (32, 42). In our assays, OZ439 nonetheless showed complete inhibition of parasite growth when assayed at 700 nM for 4 h, a concentration just below the mean peak plasma concentration (721 nM) reported after a single dose of 200 mg of OZ439 in a recent patient cohort (32). With OZ277, complete inhibition of parasite survival was never observed, with the maximum pulse concentration of 700 nM affording a 3.6% survival. In comparison, peak plasma concentrations of 112 nM to 155 nM were observed in individuals receiving either a recommended 3-day regimen of 150 mg/day OZ277 in combination with piperaquine (750 mg/day) or a 7-day course of 150 mg daily of OZ277 monotherapy (29, 31, 42).

These results corroborate a recently published study, which reported that ozonides were less effective than DHA in terms of lowering rates of parasite survival when used in short pulses (3 to 9 h) (39). The basis for the reduced activity of ozonides *in vitro* is a matter of speculation. The possibility of a faster degradation of the ozonides can be excluded, since their stability has been found in blood-stage cultures to exceed the 4-h duration of our assays. In fact, DHA was found to be the least stable compound with a loss of 50% of its activity after 8 h, whereas the stability half-life of OZ277 was 17 h and OZ439 was stable well beyond 48 h, at which time it had lost only 20% of its activity (39). DHA and ozonides share a similar pharmacophore, and their modes of action are not expected to differ substantially. It is widely accepted that the antiparasitic activity of ozonides also involves the generation of carbon-centered radicals, which lead to oxidative stress and cell damage, as has been proposed for DHA (27, 43). One possible explanation for the different potencies could be related to slower activation of the ozonides. Evidence suggests that ARTs are activated by reductive scission of the endoperoxide bond, catalyzed by free or bound iron in the parasite (35, 43). Potentially, ozonide activation might occur via the same mechanism but might be less efficient, resulting in slower parasite killing. This hypothesis is supported by a study that established a correlation between drug exposure time and activity *in vitro*. Results showed that shorter (3-h) pulses of DHA, OZ439, and OZ277 yielded a higher percentage of parasite survival, whereas longer pulses (≥6 h) completely inhibited the growth of drug-sensitive parasites (39). When using standard *in vitro* dose-response assays, which involve a 72-h-long incubation of parasites with drug (50% inhibitory concentrations [IC_50_s]), we also found no growth at concentrations as low as 12.5 nM DHA or OZ439 or 100 nM OZ277. The longer exposure to OZ439 therefore overcame any initial delay in drug activation. Under these experimental conditions, OZ439 displayed antiparasitic activities equivalent to DHA, underscoring the value of this new compound. In comparison, OZ277 exhibits mean IC_50_s 4- to 6-fold higher (17.2 nM) than either DHA (2.7 nM) or OZ439 (3.9 nM), when averaged across all wild-type-K13 parasites (see Fig. S2 and S3 and Table S2 in the supplemental material).

10.1128/mBio.00172-17.3 FIG S2  *In vitro* proliferation assays show no significant differences in IC_50_s in clinical isolates and reference lines between parasites expressing wild-type and mutant K13 for endoperoxide antimalarials. Results show IC_50_s (mean ± SEM) for DHA, OZ439, and OZ277 measured in 72-h proliferation assays with the final parasitemia determined by flow cytometry. Assays were performed on at least three separate occasions in duplicate. Results are shown for parasites expressing wild-type K13 (rev or ctrl superscript or no superscript [shown in green]), and for parasites expressing C580Y, R539T, or I543T mutant K13 (red, blue, or purple, respectively). Download FIG S2, PDF file, 0.1 MB. Copyright © 2017 Straimer et al. 2017 Straimer et al. This content is distributed under the terms of the Creative Commons Attribution 4.0 International license.

10.1128/mBio.00172-17.4 FIG S3  *In vitro* proliferation assays show no significant differences in IC_50_s between parasites expressing wild-type and mutant K13 for endoperoxide antimalarials. Results show IC_50_s (means ± SEM) for DHA, OZ439, and OZ277 measured in 72-h proliferation assays with the final parasitemia determined by flow cytometry. Assays were performed on three separate occasions in duplicate for each parasite line. Results were combined across four parasite lines harboring the wild-type *K13* allele (CamWT, Cam3.II^rev^, V1/S^ctrl^, and Cam5^rev^) and six lines carrying K13 mutations (CamWT^C580Y^, Cam3.II^C580Y^, V1/S^C580Y^, Cam3.II^R539T^, V1/S^R539T^, and Cam5^I543T^). IC_50_s were combined as follows: DHA in parasite lines expressing wild-type K13 (black) and mutant K13 (dark red), OZ439 in parasite lines expressing wild-type (dark gray) and mutant K13 (red), and OZ277 in parasite lines expressing wild-type K13 (light gray) and mutant K13 (orange). Download FIG S3, PDF file, 0.1 MB. Copyright © 2017 Straimer et al. 2017 Straimer et al. This content is distributed under the terms of the Creative Commons Attribution 4.0 International license.

10.1128/mBio.00172-17.7 TABLE S2  IC_50_s for DHA, OZ439, and OZ277. Download TABLE S2, PDF file, 0.05 MB. Copyright © 2017 Straimer et al. 2017 Straimer et al. This content is distributed under the terms of the Creative Commons Attribution 4.0 International license.

### K13 mutations mediate substantial cross-resistance to OZ277 but not OZ439.

Similarities in the modes of action of ozonides and DHA raise the concern that K13 mutations, already widespread in Asia, could immediately compromise ozonide efficacy in the field. To investigate potential cross-resistance between DHA and ozonides, we analyzed the *in vitro* susceptibility of a series of gene-edited parasite lines expressing either wild-type K13 or one of the C580Y, R539T, or I543T variants that had previously been associated with DHA resistance *in vitro* (15).

**FIG 2:**
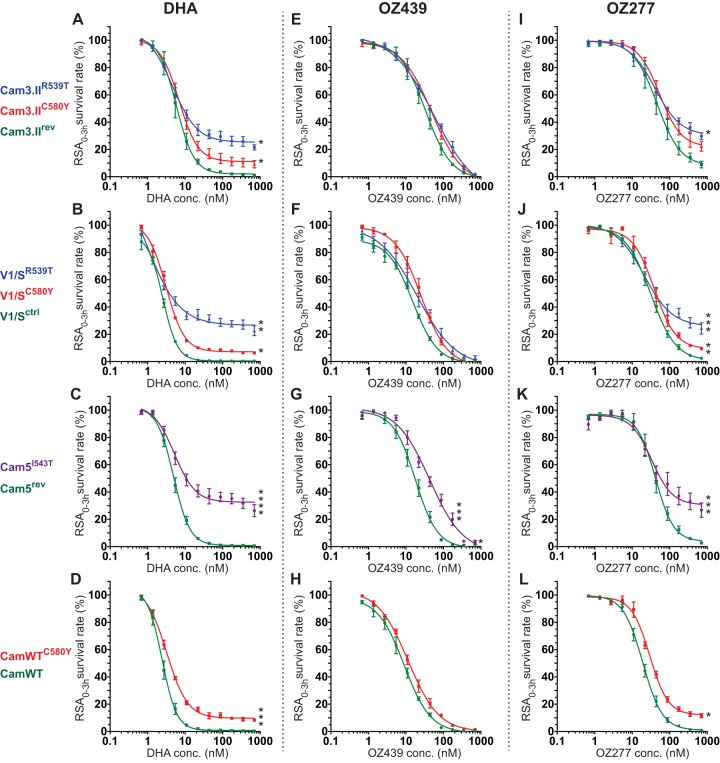
K13 propeller mutations confer cross-resistance to OZ277 but not to OZ439 in clinical isolates and reference lines *in vitro*, as defined in the ring-stage survival assay from 0 to 3 h (RSA_0–3h_). Results show the percentage of early ring-stage parasites expressing wild-type or mutant K13 (0 to 3 h postinvasion of human erythrocytes) that survive exposure to a 4-h pulse of DHA, OZ439, or OZ277 ranging in concentration from 700 nM to 0.6 nM, as measured by flow cytometry 68 h later. Data show mean ± SEM percent survival for each line assayed in duplicate on at least three independent occasions with drug or DMSO as a control. (A, E, and I) RSA_0–3h_ dose-response curves for Cam3.II parasites expressing K13 C580Y or R539T (mutation shown by the superscript) or the wild-type allele (indicated by the rev superscript). (B, F, and J) Dose-response curves for V1/S parasites expressing mutant K13 (mutation shown by the superscript) or wild-type K13 (ctrl superscript for control). (C, G, and K) Dose-response curves for Cam5 parasites expressing K13 I543T (shown in superscript) or the wild-type allele (indicated by the rev superscript). (D, H, and L) Dose-response curves for CamWT parasites expressing K13 C580Y (shown in superscript) or the wild-type allele. Student *t* tests compared percent survival values between each K13 mutant and its corresponding isogenic wild-type line, assayed for each of the four sets of parasite lines and for each drug individually when tested at 700 nM. These tests included calculations of the standard error of the difference between the means of samples being compared and the corresponding *P* values (results detailed in Table 1, with statistical outputs provided in Text S1 in the supplemental material). Cam5^I543T^ and Cam5^rev^ lines were also compared for percent survival when exposed to 175 nM OZ439. Values that are significantly different by Student *t* test are indicated as follows: *, *P* < 0.05; **, *P* < 0.01; ***, *P* < 0.001, ****, *P* < 0.0001.

Our assays documented significantly higher RSA_0–3h_ survival rates in DHA-treated parasite lines expressing mutant K13, compared to their isogenic counterparts expressing wild-type K13 (Table 1 and Fig. 2A to D). Depending on the parasite background and the *K13* allele, we observed increased survival of mutant lines at DHA concentrations as low as 2.7 nM (Fig. 2D). The survival advantage of lines expressing mutant K13 over lines expressing wild-type K13 was greater at elevated drug concentrations, where complete inhibition was never observed in the presence of mutant K13. At 700 nM DHA, the I543T mutation conferred the highest degree of resistance (26.4% in Cam5^I543T^), followed by R539T (21.5% and 22.7% in Cam3.II^R539T^ and V1/S^R539T^, respectively) and finally C580Y (9.2%, 6.4%, and 8.3% in Cam3.II^C580Y^, V1/S^C580Y^, and CamWT^C580Y^, respectively). This rank order of survival agrees with published data (15).

**TABLE 1:** Ring-stage survival assay percent survival values from drug-treated mutant-K13 or wild-type-K13 lines^a^

Parasite	700 nM DHA				700 nM OZ439				700 nM OZ277			
	RSA_0–3h_ (mean ± SEM)	*n*	*P* value^b^	Fold change^c^	RSA_0–3h_ (mean ± SEM)	*n*	*P* value	Fold change	RSA_0–3h_ (mean ± SEM)	*n*	*P* value	Fold change
Cam3.II^rev^	0.9 ± 0.3	3			1.1 ± 0.2	3			9.0 ± 2.4	3		
Cam3.II^C580Y^	9.2 ± 2.7	3	<0.05	10.5	1.4 ± 0.7	3	ns	1.2	22.3 ± 3.6	3	ns	2.5
Cam3.II^R539T^	21.5 ± 1.9	3	<0.05	24.5	1.9 ± 0.8	3	ns	1.7	29.8 ± 2.4	3	<0.05	3.3
V1/S^ctrl^	0.4 ± 0.1	3			0.7 ± 0.3	3			2.5 ± 0.4	3		
V1/S^C580Y^	6.4 ± 0.5	3	<0.05	15.0	0.5 ± 0.2	3	ns	0.7	9.4 ± 0.9	3	<0.01	3.8
V1/S^R539T^	22.6 ± 3.6	4	<0.01	53.1	2.9 ± 1.4	4	ns	4.5	24.3 ± 4.1	4	<0.001	9.9
Cam5^rev^	0.5 ± 0.1	4			0.7 ± 0.2	4			2.6 ± 0.6	4		
Cam5^I543T^	26.4 ± 4.4	4	<0.0001	51.2	3.3 ± 1.4	4	<0.05	4.6	26.7 ± 5.2	4	<0.001	10.2
CamWT	0.4 ± 0.03	3			0.5 ± 0.1	3			0.9 ± 0.3	3		
CamWT^C580Y^	8.3 ± 1.1	3	<0.001	23.6	1.0 ± 0.3	3	ns	2.1	11.6 ± 1.4	3	<0.05	13.4

^a^ RSA_0–3h_, ring-stage survival assay from 0 to 3 h; SEM, standard error of the mean; *n*, number of independent experiments.

^b^ *P* values were calculated compared to the respective reference line Cam3.II^rev^, V1/S^ctrl^, Cam5^rev^, or CamWT. ns, not significant (*P* > 0.05).

^c^ Fold increase of RSA_0–3h_ percent survival values in mutant-K13 lines compared to their respective isogenic wild-type-K13 lines.

Interestingly, OZ439 and OZ277 differed substantially in their potency against mutant-K13 ring-stage parasites in the RSA_0–3h_. OZ439 was fully effective against parasites expressing K13 C580Y or R539T in three different isolates: Cam3.II, V1/S, and CamWT (Fig. 2E, F, and H and Table 1). Our results showed reduced potency of OZ439 solely against the Cambodian parasite isolate Cam5 with the K13 I543T mutation (Cam5^I543T^), which is known to confer high levels of *in vitro* DHA resistance (compare Fig. 2C to G; Table 1). In contrast with OZ439, the activity of OZ277 was significantly impaired by all three K13 mutations analyzed in our study. We found a 2.5- to 13.4-fold increase in parasite survival depending on the K13 mutation and the parasite line (Fig. 2I to L and Table 1). At the highest pulse concentration of 700 nM, we observed an average 21.2% survival when we combined results from six parasite lines harboring one of the three mutant *K13* alleles, compared to 3.6% survival in four parasite lines carrying a wild-type *K13* allele (Fig. S4). In light of the fact that OZ277 was less potent than DHA against wild-type-K13 parasites and that a substantial number of wild-type-K13 parasites survived drug treatment, the gain of resistance in the presence of K13 mutations was also less pronounced and became significant only at higher concentrations (>87.5 nM).

10.1128/mBio.00172-17.5 FIG S4  K13 propeller mutations confer *in vitro* cross-resistance to OZ277 but not to OZ439, as defined in the ring-stage survival assay (RSA_0–3h_). Results show the percentage of early ring-stage parasites (0 to 3 h postinvasion of human erythrocytes) that survive exposure to a 4-h pulse of DHA, OZ439, or OZ277 ranging in concentration from 700 nM to 0.6 nM, as measured by flow cytometry 68 h later. Data show mean ± SEM percent survival in at least three independent experiments performed in duplicate compared with DMSO vehicle-treated parasites processed in parallel. Data show percent survival results combined across four parasite lines expressing the wild-type *K13* allele (CamWT, Cam3.II^rev^, V1/S^ctrl^, and Cam5^rev^) or six lines harboring K13 mutations (CamWT^C580Y^, Cam3.II^C580Y^, V1/S^C580Y^, Cam3.II^R539T^, V1/S^R539T^, and Cam5^I543T^). Combined RSA_0–3h_ dose-response curves are shown as follows: DHA in parasite lines expressing wild-type K13 (black squares) and mutant K13 (dark red circles), OZ439 in parasite lines expressing wild-type K13 (inverted dark gray triangles) and mutant K13 (red diamonds), and OZ277 in parasite lines expressing wild-type K13 (light gray triangles) and mutant K13 (orange squares). Student *t* tests compared percent survival values between parasite lines expressing mutant K13 and wild-type K13 exposed to a 700 nM concentration of each drug. Significance was observed for both DHA and OZ277 (****, *P* < 0.0001 for each), but not for OZ439. Download FIG S4, PDF file, 0.1 MB. Copyright © 2017 Straimer et al. 2017 Straimer et al. This content is distributed under the terms of the Creative Commons Attribution 4.0 International license.

Two recent studies also reported that OZ277 was compromised by the presence of K13 mutations (39, 44). In both studies, the RSA_0–3h_^50%^ was used to assess the susceptibility of mutant-K13 lines to ozonides and to identify potential cross-resistance with DHA. While we found this metric suitable to compare the sensitivity of wild-type-K13 parasites to different drugs, we found it less informative to evaluate mutant-K13 parasites whose dose-response curves differed in a nonconventional way between mutant-K13 and wild-type-K13 lines for all three drugs. At the lower drug concentrations, the dose-response curves overlapped between mutant-K13 and wild-type-K13 lines, whereas at the higher drug concentrations, the sensitive lines showed minimal survival, yet resistant lines reached a plateau of elevated survival that was dose independent. As an example, Cam5^rev^ and Cam5^I543T^ lines showed comparable profiles at lower concentrations, with RSA_0–3h_^50%^ values of 42.2 nM and 41.1 nM, respectively, in contrast with significantly different mean survival values of 2.6% and 26.7%, respectively, at the highest concentration of 700 nM (Fig. 2K). These altered curves were most noticeable when parasites were exposed to DHA, which presumably reflects the underlying mechanism of resistance and kinetics of the pathways involved. To demonstrate how unrepresentative the RSA_0–3h_^50%^ values were for DHA resistance *in vitro*, we compared the RSA_0–3h_^50%^ survival value to the RSA_0–3h_ survival value at 700 nM in the Cam3.II^rev^ and Cam3.II^R539T^ lines. The drug concentrations at which 50% inhibition of parasite survival was achieved were 5.8 nM and 4.6 nM, respectively, therefore revealing no major difference, whereas in contrast we observed 0.9% and 21.5% survival, respectively, in these lines exposed to 700 nM DHA. We conclude that the RSA_0–3h_^50%^ only partially reflects the ring-stage resistance phenotype (Fig. 2). Our finding that the R539T mutation in Cam3.II parasites does not mediate cross-resistance to OZ439 agrees with two other reports (44, 45), whereas a third observed cross-resistance (39). Of note, our R539T data were consistent in both genetic backgrounds tested (Cam3.II and V1/S), and our study extends these earlier reports by assessing the roles of K13 mutations across four distinct genetic backgrounds and three mutant-K13 isoforms compared to isogenic wild-type-K13 lines.

Our finding that K13 mutations mediate significant cross-resistance to the first-generation endoperoxide OZ277 is of major concern (Fig. 2I, J, K, and L). Another concern with this short plasma half-life drug is its lack of accumulation in malaria patients even over the course of a recommended 7-day treatment of 150 mg daily (31). In those studies, the mean/peak plasma concentration reached only ~120 nM, which is significantly below the maximal concentration used in our assay and which we observed to be insufficient to inhibit the survival of parasites with wild-type or mutant K13. Its recent registration in India as a licensed drug, in combination with piperaquine, might therefore result in selection for K13 mutants that could compromise both OZ277 and ART efficacy. Indeed, a very recent study from India has detected K13 mutations (notably F446I associated with resistance in Myanmar) (46). OZ277 resistance would in turn place selective pressure on piperaquine, to which resistance is currently spreading in Cambodia on K13 mutant genetic backgrounds, resulting in high levels of treatment failure (47, 48). Monitoring the K13 status and clearance half-lives of Indian infections treated with the combination of OZ277 and piperaquine will therefore be important.

Our findings also provide insight into the suitability of OZ439 as an alternative treatment option, including in Southeast Asia where ACT treatment failures are increasing (1). Studies suggest that its longer half-life (compared to DHA) should suffice to eliminate mutant-K13 parasites in patients, based on cumulative effective doses modeled in Cambodian lines expressing wild-type or R539T K13 (39). Our data provide evidence of nearly equivalent efficacies of OZ439 against mutant-K13 and wild-type-K13 isogenic lines developed on four separate genetic backgrounds, with the exception of the I543T mutation that conferred a degree of protection in the survival assay (although full clearance was nonetheless achieved at high concentrations; Fig. 2G). These data support the ongoing development of OZ439, combined with close scrutiny to test for any selection favoring mutant-K13 parasites, including ones harboring I543T.

### K13 mutations differ in their impact on parasite growth rates *in vitro*.

Earlier studies with the primary chloroquine resistance determinant PfCRT (*P. falciparum* chloroquine resistance transporter) have shown that fitness costs associated with mutant alleles substantially impacted the prevalence and dissemination of chloroquine-resistant strains and favored alleles with the least fitness defect. Whereas extensive chloroquine use led to a selective sweep of mutant *pfcrt* worldwide, its subsequent removal resulted in less-fit mutant parasites being overtaken by ones expressing wild-type *pfcrt* (42, 49–51). Fitness costs were also observed in an ART drug-pressured mutant *P. falciparum* line, although the molecular basis of resistance was unrelated to K13 and has not been defined (52). We hypothesized that in addition to the broad spectrum of resistance levels conferred by the different *K13* alleles, fitness costs conveyed by these mutations may also play an essential role in establishing the viability and distribution of a given haplotype in settings where malaria is endemic. To investigate parasite fitness, we compared *in vitro* growth rates in competition experiments using isogenic parasite pairs differing solely in their *K13* allele status (wild type versus mutant). Since C580Y is the most prevalent allele in Southeast Asia, we selected three different parasite lines carrying this mutation, engineered into two recent Cambodian isolates (Cam3.II and CamWT) as well as the older V1/S line that was culture adapted (in 1976) well before ART was widely deployed clinically. Furthermore, we chose the Cam3.II and V1/S lines that expressed the R539T mutation and extended the study to the I543T mutation expressed in Cam5 parasites.

**FIG 3:**
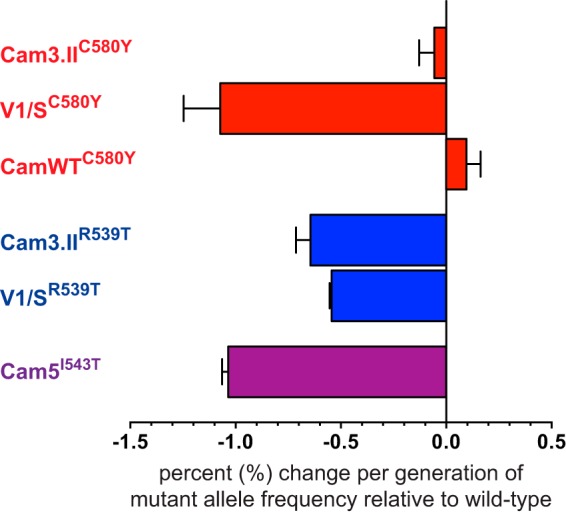
K13 mutations confer an *in vitro* fitness cost in clinical isolates and reference lines. Results show differences in growth rates per 48-h generation of clinical isolates and reference lines harboring native or ZFN-edited K13 mutations (shown in superscript) relative to their isogenic parasite lines carrying the wild-type *K13* allele (e.g., Cam3.II^C580Y^ versus Cam3.II^rev^, showing a mean 0.06% reduction in the rate of growth of the C580Y mutant relative to its isogenic K13 wild-type control). Differences in growth rates were calculated as the percent change in *K13* mutant allele frequency over a 60-day coculture period, as determined by pyrosequencing. Values are shown as means ± SEM (error bars) in two independent assays performed in duplicate.

Growth competition assays were initiated as mixed cultures of isogenic wild-type and mutant parasites in a 1:1 ratio. Genomic DNA was collected every 3 to 4 days over a period of 60 days, and allele frequency was determined via pyrosequencing. This allowed us to calculate the percent changes per 48-h generation of the mutant allele compared to the wild-type allele (Fig. 3). Results showed variable growth defects exerted by K13 mutations on mutant parasites compared to their wild-type-K13 counterparts, with results evoking an important contribution of the parasite genetic background.

These fitness costs were most pronounced in parasites expressing K13 R539T or I543T in comparison with their isogenic wild-type-K13 lines (−0.6% in Cam3.II^R539T^, −0.5% in V1/S^R539T^, and −1.0% in Cam5^I543T^). These two mutations have previously been observed to confer high levels of *in vitro* drug resistance and significantly prolonged clearance times in patients (10, 13, 15). In contrast, the fitness deficit conveyed by the C580Y allele was essentially nil in two recently culture-adapted Cambodian isolates (−0.06% in Cam3.II^C580Y^ and +0.1% in CamWT^C580Y^). The earlier demonstration that C580Y exhibits lower *in vitro* resistance (15) highlights the fact that RSA_0–3h_ survival rates alone are not sufficient to predict the spread of individual K13 mutations and evoke an important role for fitness. Our findings are also consistent with the expansion and fixation of this mutation in some parts of Southeast Asia (12, 22, 53). Our evidence that C580Y is the fittest *K13* allele is further corroborated by a direct growth competition assay of C580Y with R539T in the Cam3.II background, in which we observed a greater fitness deficit (−0.4%) in parasites expressing K13 R539T compared to an isogenic K13 C580Y line.

In contrast to its lack of a fitness cost in the Cam3.II and CamWT backgrounds, the C580Y mutation in the V1/S strain showed a marked growth defect *in vitro* (−1.1% per 48-h generation) compared to its isogenic wild-type-K13 counterpart (Fig. 3). This suggests an important contribution of the parasite genetic background, which would render the recent Cambodian isolates more receptive to the evolution of ART resistance than older reference lines. These findings support recent population genetics-based studies, which found that resistance to ART evolved *de novo* on multiple occasions within several “founder” populations present in Cambodia and Vietnam (13, 18, 54, 55). Interestingly, ART-resistant founder populations have been observed to also contain a suite of additional single nucleotide polymorphisms (SNPs) that are in linkage disequilibrium with *K13* (14, 56), suggesting a role in either facilitating or augmenting resistance or enhancing fitness or both (23, 57). Further studies will be required to address other aspects of parasite fitness beyond asexual blood-stage growth rates, notably whether K13 mutations impact gametocytogenesis and transmission to the *Anopheles* vector. Our data argue that C580Y in particular poses a minimal fitness deficit in its impact on blood-stage proliferation and reinforce the need to monitor the appearance of K13 mutations worldwide and assess their impact on ART efficacy.

## MATERIALS AND METHODS

### Parasite lines.

The parasite lines used herein were described previously (15), with the exception of V1/S^C580Y^ that was developed for this study. In brief, zinc-finger nucleases (ZFNs) directed against *K13* (PlasmoDB identifier [ID] PF3D7_1343700, also known as *Kelch13*) were designed and leveraged to introduce either the wild-type *K13* allele or one of three mutant alleles: C580Y, R539T, or I543T. Gene editing was identified by PCR, and clones were obtained by limiting dilution and confirmed by PCR and sequence analysis, as previously described (15).

### Parasite cultures.

Asexual blood-stage parasites were propagated in human erythrocytes in RPMI 1640 malaria culture medium containing 2 mM l-glutamine, 50 mg/liter hypoxanthine, 25 mM HEPES, 0.225% NaHCO_3_, 10 mg/liter gentamicin, and 0.5% (wt/vol) Albumax II (Invitrogen). Parasites were maintained at 37°C under an atmosphere of 5% O_2_, 5% CO_2_, and 90% N_2_.

### Ring-stage survival assays (RSA_0–3h_).

The ring-stage survival assays (ring-stage survival assay from 0 to 3 h [RSA_0–3h_]) were carried out as previously described (15, 40), with minor modifications. In brief, parasite cultures were synchronized 1 or 2 times using 5% sorbitol (Sigma-Aldrich). Synchronous schizonts were incubated for 30 min at 37°C in RPMI 1640 medium supplemented with 25 mM HEPES and 15 U/ml sodium heparin. Parasites were concentrated over a gradient of 75% Percoll (Sigma-Aldrich), washed once in RPMI 1640 medium, supplemented with 25 mM HEPES, and incubated for 3 h with fresh erythrocytes in complete culture medium to allow time for merozoite invasion. The cultures were again treated with sorbitol to eliminate remaining schizonts. The 0- to 3-h postinvasion rings were adjusted to 1% parasitemia and 1% hematocrit and exposed to a range of concentrations of DHA, OZ439, OZ277 (0.68 to 700 nM), or 0.1% dimethyl sulfoxide (DMSO) (solvent control) for 4 h in 96-well plates with 200-µl cultures/well. Previously, it had been demonstrated that incomplete removal of the drug caused by insufficient washing following the 4-h incubation could result in overestimating drug activity (39). Therefore, we employed a stringent washing procedure involving four consecutive washes with 200 µl of incomplete medium and two transfers of cells to new 96-well plates to ensure complete removal of the drug, as recommended (39). Parasites were then returned to standard culture conditions for an additional 66 h. Parasite survival was assessed by staining with 2× SYBR green I and 165 nM MitoTracker deep red (Invitrogen). Parasitemia was determined by flow cytometry on an Accuri C6 cytometer (58). Between 60,000 and 100,000 cells were examined for each data point. After 72 h, parasitemias had generally expanded to 3 to 5% in DMSO-treated controls, which were used as the reference to calculate the percent survival of drug-treated parasites (40).

### *In vitro* IC_50_ assays.

*In vitro* 50% inhibitory concentrations (IC_50_s) were determined by incubating parasites for 72 h across a range of concentrations of DHA (0.048 to 100 nM), OZ439 (0.028 to 207.6 nM), or OZ277 (0.39 to 800 nM). Proliferation was determined by flow cytometry (see above). *In vitro* IC_50_s were calculated by nonlinear regression analysis, and Student *t* tests were used for statistical analysis.

### *In vitro* mixed-culture competition assays, pyrosequencing, and determination of relative growth rates.

For growth competition assays, two parasite lines were mixed 1:1 and seeded in duplicate at an initial parasitemia of 3% ring-stage parasites in drug-free medium. Parasitemia was maintained between 0.3% and 8% to ensure optimal growth conditions. Two to four separate competition assays were performed in duplicate for each parasite pair, and each assay was monitored for an average of 60 days. To determine the ratio of both strains in the mixture over time, saponin-lysed parasite pellets of the mixed cultures were collected on average every 3 or 4 days, and DNA was extracted using DNeasy blood and tissue kits (Qiagen). DNAs were used to determine individual allele frequencies in these mixed cultures by pyrosequencing codon 539, 543, or 580 (see Table S3** **in the supplemental material). To calculate the relative growth rates of individual parasite lines, the relative proportion of the two distinct *K13* alleles (whose values were always between 0 and 1) were natural log transformed, and linear regression was applied to estimate the relative growth rate value.

10.1128/mBio.00172-17.8 TABLE S3  Oligonucleotides used in this study. Download TABLE S3, PDF file, 0.04 MB. Copyright © 2017 Straimer et al. 2017 Straimer et al. This content is distributed under the terms of the Creative Commons Attribution 4.0 International license.

### Parasite availability.

Parental and transgenic parasite lines are available through BEI Resources (www.beiresources.org) or upon request from D. A. Fidock.[Supplementary-material textS1]

10.1128/mBio.00172-17.1 TEXT S1  Supplemental statistics. Download TEXT S1, DOCX file, 1.2 MB. Copyright © 2017 Straimer et al. 2017 Straimer et al. This content is distributed under the terms of the Creative Commons Attribution 4.0 International license.

## References

[1] World Health Organization 2016 Artemisinin and artemisinin-based combination therapy resistance. WHO reference number WHO/HTM/GMP/2016.11 Global Malaria Programme, World Health Organization, Geneva, Switzerland http://www.who.int/malaria/publications/atoz/update-artemisinin-resistance-october2016/en.

[2] Woodrow CJ, White NJ 2017 The clinical impact of artemisinin resistance in Southeast Asia and the potential for future spread. FEMS Microbiol Rev 41:34–48. doi:10.1093/femsre/fuw037. 27613271 PMC5424521

[3] White NJ 2008 Qinghaosu (artemisinin): the price of success. Science 320:330–334. doi:10.1126/science.1155165. 18420924

[4] Dondorp AM, Nosten F, Yi P, Das D, Phyo AP, Tarning J, Lwin KM, Ariey F, Hanpithakpong W, Lee SJ, Ringwald P, Silamut K, Imwong M, Chotivanich K, Lim P, Herdman T, An SS, Yeung S, Singhasivanon P, Day NP, Lindegardh N, Socheat D, White NJ 2009 Artemisinin resistance in *Plasmodium falciparum* malaria. N Engl J Med 361:455–467. doi:10.1056/NEJMoa0808859. 19641202 PMC3495232

[5] Noedl H, Se Y, Schaecher K, Smith BL, Socheat D, Fukuda MM, Artemisinin Resistance in Cambodia 1 (ARC1) Study Consortium 2008 Evidence of artemisinin-resistant malaria in western Cambodia. N Engl J Med 359:2619–2620. doi:10.1056/NEJMc0805011. 19064625

[6] Rogers WO, Sem R, Tero T, Chim P, Lim P, Muth S, Socheat D, Ariey F, Wongsrichanalai C 2009 Failure of artesunate-mefloquine combination therapy for uncomplicated *Plasmodium falciparum* malaria in southern Cambodia. Malar J 8:10. doi:10.1186/1475-2875-8-10. 19138388 PMC2628668

[7] Amaratunga C, Lim P, Suon S, Sreng S, Mao S, Sopha C, Sam B, Dek D, Try V, Amato R, Blessborn D, Song L, Tullo GS, Fay MP, Anderson JM, Tarning J, Fairhurst RM 2016 Dihydroartemisinin-piperaquine resistance in *Plasmodium falciparum* malaria in Cambodia: a multisite prospective cohort study. Lancet Infect Dis 16:357–365. doi:10.1016/S1473-3099(15)00487-9. 26774243 PMC4792715

[8] Saunders DL, Vanachayangkul P, Lon C, US Army Military Malaria Research Program, National Center for Parasitology, Entomology, and Malaria Control (CNM), Royal Cambodian Armed Forces 2014 Dihydroartemisinin-piperaquine failure in Cambodia. N Engl J Med 371:484–485. doi:10.1056/NEJMc1403007. 25075853

[9] Spring MD, Lin JT, Manning JE, Vanachayangkul P, Somethy S, Bun R, Se Y, Chann S, Ittiverakul M, Sia-ngam P, Kuntawunginn W, Arsanok M, Buathong N, Chaorattanakawee S, Gosi P, Ta-aksorn W, Chanarat N, Sundrakes S, Kong N, Heng TK, Nou S, Teja-isavadharm P, Pichyangkul S, Phann ST, Balasubramanian S, Juliano JJ, Meshnick SR, Chour CM, Prom S, Lanteri CA, Lon C, Saunders DL 2015 Dihydroartemisinin-piperaquine failure associated with a triple mutant including kelch13 C580Y in Cambodia: an observational cohort study. Lancet Infect Dis 15:683–691. doi:10.1016/S1473-3099(15)70049-6. 25877962

[10] Ariey F, Witkowski B, Amaratunga C, Beghain J, Langlois AC, Khim N, Kim S, Duru V, Bouchier C, Ma L, Lim P, Leang R, Duong S, Sreng S, Suon S, Chuor CM, Bout DM, Ménard S, Rogers WO, Genton B, Fandeur T, Miotto O, Ringwald P, Le Bras J, Berry A, Barale JC, Fairhurst RM, Benoit-Vical F, Mercereau-Puijalon O, Ménard D 2014 A molecular marker of artemisinin-resistant *Plasmodium falciparum* malaria. Nature 505:50–55. doi:10.1038/nature12876. 24352242 PMC5007947

[11] Takala-Harrison S, Clark TG, Jacob CG, Cummings MP, Miotto O, Dondorp AM, Fukuda MM, Nosten F, Noedl H, Imwong M, Bethell D, Se Y, Lon C, Tyner SD, Saunders DL, Socheat D, Ariey F, Phyo AP, Starzengruber P, Fuehrer HP, Swoboda P, Stepniewska K, Flegg J, Arze C, Cerqueira GC, Silva JC, Ricklefs SM, Porcella SF, Stephens RM, Adams M, Kenefic LJ, Campino S, Auburn S, MacInnis B, Kwiatkowski DP, Su XZ, White NJ, Ringwald P, Plowe CV 2013 Genetic loci associated with delayed clearance of *Plasmodium falciparum* following artemisinin treatment in Southeast Asia. Proc Natl Acad Sci U S A 110:240–245. doi:10.1073/pnas.1211205110. 23248304 PMC3538248

[12] Ménard D, Khim N, Beghain J, Adegnika AA, Shafiul-Alam M, Amodu O, Rahim-Awab G, Barnadas C, Berry A, Boum Y, Bustos MD, Cao J, Chen JH, Collet L, Cui L, Thakur GD, Dieye A, Djallé D, Dorkenoo MA, Eboumbou-Moukoko CE, Espino FE, Fandeur T, Ferreira-da-Cruz MF, Fola AA, Fuehrer HP, Hassan AM, Herrera S, Hongvanthong B, Houzé S, Ibrahim ML, Jahirul-Karim M, Jiang L, Kano S, Ali-Khan W, Khanthavong M, Kremsner PG, Lacerda M, Leang R, Leelawong M, Li M, Lin K, Mazarati JB, Menard S, Morlais I, Muhindo-Mavoko H, Musset L, Na-Bangchang K, Nambozi M, Niare K, Noedl H, Ouédraogo JB, et al. 2016 A worldwide map of *Plasmodium falciparum* K13-propeller polymorphisms. N Engl J Med 374:2453–2464. doi:10.1056/NEJMoa1513137. 27332904 PMC4955562

[13] Takala-Harrison S, Jacob CG, Arze C, Cummings MP, Silva JC, Dondorp AM, Fukuda MM, Hien TT, Mayxay M, Noedl H, Nosten F, Kyaw MP, Nhien NT, Imwong M, Bethell D, Se Y, Lon C, Tyner SD, Saunders DL, Ariey F, Mercereau-Puijalon O, Menard D, Newton PN, Khanthavong M, Hongvanthong B, Starzengruber P, Fuehrer HP, Swoboda P, Khan WA, Phyo AP, Nyunt MM, Nyunt MH, Brown TS, Adams M, Pepin CS, Bailey J, Tan JC, Ferdig MT, Clark TG, Miotto O, MacInnis B, Kwiatkowski DP, White NJ, Ringwald P, Plowe CV 2015 Independent emergence of artemisinin resistance mutations among *Plasmodium falciparum* in Southeast Asia. J Infect Dis 211:670–679. doi:10.1093/infdis/jiu491. 25180241 PMC4334802

[14] Miotto O, Amato R, Ashley EA, MacInnis B, Almagro-Garcia J, Amaratunga C, Lim P, Mead D, Oyola SO, Dhorda M, Imwong M, Woodrow C, Manske M, Stalker J, Drury E, Campino S, Amenga-Etego L, Thanh TN, Tran HT, Ringwald P, Bethell D, Nosten F, Phyo AP, Pukrittayakamee S, Chotivanich K, Chuor CM, Nguon C, Suon S, Sreng S, Newton PN, Mayxay M, Khanthavong M, Hongvanthong B, Htut Y, Han KT, Kyaw MP, Faiz MA, Fanello CI, Onyamboko M, Mokuolu OA, Jacob CG, Takala-Harrison S, Plowe CV, Day NP, Dondorp AM, Spencer CC, McVean G, Fairhurst RM, White NJ, Kwiatkowski DP 2015 Genetic architecture of artemisinin-resistant *Plasmodium falciparum*. Nat Genet 47:226–234. doi:10.1038/ng.3189. 25599401 PMC4545236

[15] Straimer J, Gnädig NF, Witkowski B, Amaratunga C, Duru V, Ramadani AP, Dacheux M, Khim N, Zhang L, Lam S, Gregory PD, Urnov FD, Mercereau-Puijalon O, Benoit-Vical F, Fairhurst RM, Ménard D, Fidock DA 2015 K13-propeller mutations confer artemisinin resistance in *Plasmodium falciparum* clinical isolates. Science 347:428–431. doi:10.1126/science.1260867. 25502314 PMC4349400

[16] Ghorbal M, Gorman M, Macpherson CR, Martins RM, Scherf A, Lopez-Rubio JJ 2014 Genome editing in the human malaria parasite *Plasmodium falciparum* using the CRISPR-Cas9 system. Nat Biotechnol 32:819–821. doi:10.1038/nbt.2925. 24880488

[17] Tun KM, Imwong M, Lwin KM, Win AA, Hlaing TM, Hlaing T, Lin K, Kyaw MP, Plewes K, Faiz MA, Dhorda M, Cheah PY, Pukrittayakamee S, Ashley EA, Anderson TJ, Nair S, McDew-White M, Flegg JA, Grist EP, Guerin P, Maude RJ, Smithuis F, Dondorp AM, Day NP, Nosten F, White NJ, Woodrow CJ 2015 Spread of artemisinin-resistant *Plasmodium falciparum* in Myanmar: a cross-sectional survey of the K13 molecular marker. Lancet Infect Dis 15:415–421. doi:10.1016/S1473-3099(15)70032-0. 25704894 PMC4374103

[18] Talundzic E, Okoth SA, Congpuong K, Plucinski MM, Morton L, Goldman IF, Kachur PS, Wongsrichanalai C, Satimai W, Barnwell JW, Udhayakumar V 2015 Selection and spread of artemisinin-resistant alleles in Thailand prior to the global artemisinin resistance containment campaign. PLoS Pathog 11:e1004789. doi:10.1371/journal.ppat.1004789. 25836766 PMC4383523

[19] Bosman P, Stassijns J, Nackers F, Canier L, Kim N, Khim S, Alipon SC, Chuor Char M, Chea N, Dysoley L, Van den Bergh R, Etienne W, De Smet M, Ménard D, Kindermans JM 2014 *Plasmodium* prevalence and artemisinin-resistant falciparum malaria in Preah Vihear Province, Cambodia: a cross-sectional population-based study. Malar J 13:394. doi:10.1186/1475-2875-13-394. 25288380 PMC4200124

[20] Leang R, Taylor WR, Bouth DM, Song L, Tarning J, Char MC, Kim S, Witkowski B, Duru V, Domergue A, Khim N, Ringwald P, Menard D 2015 Evidence of *Plasmodium falciparum* malaria multidrug resistance to artemisinin and piperaquine in western Cambodia: dihydroartemisinin-piperaquine open-label multicenter clinical assessment. Antimicrob Agents Chemother 59:4719–4726. doi:10.1128/AAC.00835-15. 26014949 PMC4505193

[21] Imwong M, Jindakhad T, Kunasol C, Sutawong K, Vejakama P, Dondorp AM 2015 An outbreak of artemisinin resistant falciparum malaria in Eastern Thailand. Sci Rep 5:17412. doi:10.1038/srep17412. 26616851 PMC4663761

[22] MalariaGEN *Plasmodium falciparum* Community Project 2016 Genomic epidemiology of artemisinin resistant malaria. Elife 5:e08714. doi:10.7554/eLife.08714. 26943619 PMC4786412

[23] Rosenthal PJ 2013 The interplay between drug resistance and fitness in malaria parasites. Mol Microbiol 89:1025–1038. doi:10.1111/mmi.12349. 23899091 PMC3792794

[24] Wells TN, Hooft van Huijsduijnen R, Van Voorhis WC 2015 Malaria medicines: a glass half full? Nat Rev Drug Discov 14:424–442. doi:10.1038/nrd4573. 26000721

[25] Leroy D 2017 How to tackle antimalarial resistance? EMBO Mol Med 9:133–134. doi:10.15252/emmm.201607295. 28028013 PMC5286390

[26] Kaiser M, Wittlin S, Nehrbass-Stuedli A, Dong Y, Wang X, Hemphill A, Matile H, Brun R, Vennerstrom JL 2007 Peroxide bond-dependent antiplasmodial specificity of artemisinin and OZ277 (RBx11160). Antimicrob Agents Chemother 51:2991–2993. doi:10.1128/AAC.00225-07. 17562801 PMC1932508

[27] Fügi MA, Wittlin S, Dong Y, Vennerstrom JL 2010 Probing the antimalarial mechanism of artemisinin and OZ277 (arterolane) with nonperoxidic isosteres and nitroxyl radicals. Antimicrob Agents Chemother 54:1042–1046. doi:10.1128/AAC.01305-09. 20028825 PMC2825978

[28] Charman SA, Arbe-Barnes S, Bathurst IC, Brun R, Campbell M, Charman WN, Chiu FC, Chollet J, Craft JC, Creek DJ, Dong Y, Matile H, Maurer M, Morizzi J, Nguyen T, Papastogiannidis P, Scheurer C, Shackleford DM, Sriraghavan K, Stingelin L, Tang Y, Urwyler H, Wang X, White KL, Wittlin S, Zhou L, Vennerstrom JL 2011 Synthetic ozonide drug candidate OZ439 offers new hope for a single-dose cure of uncomplicated malaria. Proc Natl Acad Sci U S A 108:4400–4405. doi:10.1073/pnas.1015762108. 21300861 PMC3060245

[29] Saha N, Moehrle JJ, Zutshi A, Sharma P, Kaur P, Iyer SS 2014 Safety, tolerability and pharmacokinetic profile of single and multiple oral doses of arterolane (RBx11160) maleate in healthy subjects. J Clin Pharmacol 54:386–393. doi:10.1002/jcph.232. 24242999

[30] Gautam A, Ahmed T, Sharma P, Varshney B, Kothari M, Saha N, Roy A, Moehrle JJ, Paliwal J 2011 Pharmacokinetics and pharmacodynamics of arterolane maleate following multiple oral doses in adult patients with *P. falciparum* malaria. J Clin Pharmacol 51:1519–1528. doi:10.1177/0091270010385578. 21148048

[31] Valecha N, Krudsood S, Tangpukdee N, Mohanty S, Sharma SK, Tyagi PK, Anvikar A, Mohanty R, Rao BS, Jha AC, Shahi B, Singh JP, Roy A, Kaur P, Kothari M, Mehta S, Gautam A, Paliwal JK, Arora S, Saha N 2012 Arterolane maleate plus piperaquine phosphate for treatment of uncomplicated *Plasmodium falciparum* malaria: a comparative, multicenter, randomized clinical trial. Clin Infect Dis 55:663–671. doi:10.1093/cid/cis475. 22586253

[32] Phyo AP, Jittamala P, Nosten FH, Pukrittayakamee S, Imwong M, White NJ, Duparc S, Macintyre F, Baker M, Möhrle JJ 2016 Antimalarial activity of artefenomel (OZ439), a novel synthetic antimalarial endoperoxide, in patients with *Plasmodium falciparum* and *Plasmodium vivax* malaria: an open-label phase 2 trial. Lancet Infect Dis 16:61–69. doi:10.1016/S1473-3099(15)00320-5. 26448141 PMC4700386

[33] Moehrle JJ, Duparc S, Siethoff C, van Giersbergen PL, Craft JC, Arbe-Barnes S, Charman SA, Gutierrez M, Wittlin S, Vennerstrom JL 2013 First-in-man safety and pharmacokinetics of synthetic ozonide OZ439 demonstrates an improved exposure profile relative to other peroxide antimalarials. Br J Clin Pharmacol 75:524–537. doi:10.1111/j.1365-2125.2012.04368.x. 22759078 PMC3558805

[34] Meunier B, Robert A 2010 Heme as trigger and target for trioxane-containing antimalarial drugs. Acc Chem Res 43:1444–1451. doi:10.1021/ar100070k. 20804120

[35] Meshnick SR 2002 Artemisinin: mechanisms of action, resistance and toxicity. Int J Parasitol 32:1655–1660. doi:10.1016/S0020-7519(02)00194-7. 12435450

[36] Klonis N, Creek DJ, Tilley L 2013 Iron and heme metabolism in *Plasmodium falciparum* and the mechanism of action of artemisinins. Curr Opin Microbiol 16:722–727. doi:10.1016/j.mib.2013.07.005. 23932203

[37] Tilley L, Straimer J, Gnädig NF, Ralph SA, Fidock DA 2016 Artemisinin action and resistance in *Plasmodium falciparum*. Trends Parasitol 32:682–696. doi:10.1016/j.pt.2016.05.010. 27289273 PMC5007624

[38] Delves M, Plouffe D, Scheurer C, Meister S, Wittlin S, Winzeler EA, Sinden RE, Leroy D 2012 The activities of current antimalarial drugs on the life cycle stages of *Plasmodium*: a comparative study with human and rodent parasites. PLoS Med 9:e1001169. doi:10.1371/journal.pmed.1001169. 22363211 PMC3283556

[39] Yang T, Xie SC, Cao P, Giannangelo C, McCaw J, Creek DJ, Charman SA, Klonis N, Tilley L 2016 Comparison of the exposure time dependence of the activities of synthetic ozonide antimalarials and dihydroartemisinin against K13 wild-type and mutant *Plasmodium falciparum* strains. Antimicrob Agents Chemother 60:4501–4510. doi:10.1128/AAC.00574-16. 27161632 PMC4958167

[40] Witkowski B, Amaratunga C, Khim N, Sreng S, Chim P, Kim S, Lim P, Mao S, Sopha C, Sam B, Anderson JM, Duong S, Chuor CM, Taylor WR, Suon S, Mercereau-Puijalon O, Fairhurst RM, Menard D 2013 Novel phenotypic assays for the detection of artemisinin-resistant *Plasmodium falciparum* malaria in Cambodia: *in-vitro* and *ex-vivo* drug-response studies. Lancet Infect Dis 13:1043–1049. doi:10.1016/S1473-3099(13)70252-4. 24035558 PMC5015432

[41] Ashley EA, Dhorda M, Fairhurst RM, Amaratunga C, Lim P, Suon S, Sreng S, Anderson JM, Mao S, Sam B, Sopha C, Chuor CM, Nguon C, Sovannaroth S, Pukrittayakamee S, Jittamala P, Chotivanich K, Chutasmit K, Suchatsoonthorn C, Runcharoen R, Hien TT, Thuy-Nhien NT, Thanh NV, Phu NH, Htut Y, Han KT, Aye KH, Mokuolu OA, Olaosebikan RR, Folaranmi OO, Mayxay M, Khanthavong M, Hongvanthong B, Newton PN, Onyamboko MA, Fanello CI, Tshefu AK, Mishra N, Valecha N, Phyo AP, Nosten F, Yi P, Tripura R, Borrmann S, Bashraheil M, Peshu J, Faiz MA, Ghose A, Hossain MA, Samad R, Rahman MR, et al. 2014 Spread of artemisinin resistance in *Plasmodium falciparum* malaria. N Engl J Med 371:411–423. doi:10.1056/NEJMoa1314981. 25075834 PMC4143591

[42] Valecha N, Looareesuwan S, Martensson A, Abdulla SM, Krudsood S, Tangpukdee N, Mohanty S, Mishra SK, Tyagi PK, Sharma SK, Moehrle J, Gautam A, Roy A, Paliwal JK, Kothari M, Saha N, Dash AP, Björkman A 2010 Arterolane, a new synthetic trioxolane for treatment of uncomplicated *Plasmodium falciparum* malaria: a phase II, multicenter, randomized, dose-finding clinical trial. Clin Infect Dis 51:684–691. doi:10.1086/655831. 20687837

[43] O’Neill PM, Barton VE, Ward SA 2010 The molecular mechanism of action of artemisinin—the debate continues. Molecules 15:1705–1721. doi:10.3390/molecules15031705. 20336009 PMC6257357

[44] Siriwardana A, Iyengar K, Roepe PD 2016 Endoperoxide drug cross resistance patterns for *Plasmodium falciparum* exhibiting an artemisinin delayed clearance phenotype. Antimicrob Agents Chemother 60:6952–6956. doi:10.1128/AAC.00857-16. 27600038 PMC5075116

[45] Baumgärtner F, Jourdan J, Scheurer C, Blasco B, Campo B, Mäser P, Wittlin S 2017 *In vitro* activity of anti-malarial ozonides against an artemisinin-resistant isolate. Malar J 16:45. doi:10.1186/s12936-017-1696-0. 28122617 PMC5267415

[46] Mishra N, Bharti RS, Mallick P, Singh OP, Srivastava B, Rana R, Phookan S, Gupta HP, Ringwald P, Valecha N 2016 Emerging polymorphisms in falciparum Kelch 13 gene in Northeastern region of India. Malar J 15:583. doi:10.1186/s12936-016-1636-4. 27912758 PMC5135801

[47] Amato R, Lim P, Miotto O, Amaratunga C, Dek D, Pearson RD, Almagro-Garcia J, Neal AT, Sreng S, Suon S, Drury E, Jyothi D, Stalker J, Kwiatkowski DP, Fairhurst RM 2017 Genetic markers associated with dihydroartemisinin-piperaquine failure in *Plasmodium falciparum* malaria in Cambodia: a genotype-phenotype association study. Lancet Infect Dis 17:164–173. doi:10.1016/S1473-3099(16)30409-1. 27818095 PMC5564489

[48] Witkowski B, Duru V, Khim N, Ross LS, Saintpierre B, Beghain J, Chy S, Kim S, Ke S, Kloeung N, Eam R, Khean C, Ken M, Loch K, Bouillon A, Domergue A, Ma L, Bouchier C, Leang R, Huy R, Nuel G, Barale JC, Legrand E, Ringwald P, Fidock DA, Mercereau-Puijalon O, Ariey F, Ménard D 2017 A surrogate marker of piperaquine-resistant *Plasmodium falciparum* malaria: a phenotype-genotype association study. Lancet Infect Dis 17:174–183. doi:10.1016/S1473-3099(16)30415-7. 27818097 PMC5266792

[49] Laufer MK, Takala-Harrison S, Dzinjalamala FK, Stine OC, Taylor TE, Plowe CV 2010 Return of chloroquine-susceptible falciparum malaria in Malawi was a reexpansion of diverse susceptible parasites. J Infect Dis 202:801–808. doi:10.1086/655659. 20662717 PMC3380613

[50] Gabryszewski SJ, Dhingra SK, Combrinck JM, Lewis IA, Callaghan PS, Hassett MR, Siriwardana A, Henrich PP, Lee AH, Gnädig NF, Musset L, Llinás M, Egan TJ, Roepe PD, Fidock DA 2016 Evolution of fitness cost-neutral mutant PfCRT conferring *P. falciparum* 4-aminoquinoline drug resistance is accompanied by altered parasite metabolism and digestive vacuole physiology. PLoS Pathog 12:e1005976. doi:10.1371/journal.ppat.1005976. 27832198 PMC5104409

[51] Gabryszewski SJ, Modchang C, Musset L, Chookajorn T, Fidock DA 2016 Combinatorial genetic modeling of *pfcrt*-mediated drug resistance evolution in *Plasmodium falciparum*. Mol Biol Evol 33:1554–1570. doi:10.1093/molbev/msw037. 26908582 PMC4868112

[52] Hott A, Tucker MS, Casandra D, Sparks K, Kyle DE 2015 Fitness of artemisinin-resistant *Plasmodium falciparum* *in* *vitro*. J Antimicrob Chemother 70:2787–2796. doi:10.1093/jac/dkv199. 26203183 PMC4668880

[53] Anderson TJ, Nair S, McDew-White M, Cheeseman IH, Nkhoma S, Bilgic F, McGready R, Ashley E, Pyae Phyo A, White NJ, Nosten F 2017 Population parameters underlying an ongoing soft sweep in Southeast Asian malaria parasites. Mol Biol Evol 34:131–144. doi:10.1093/molbev/msw228. 28025270 PMC5216669

[54] Miotto O, Almagro-Garcia J, Manske M, Macinnis B, Campino S, Rockett KA, Amaratunga C, Lim P, Suon S, Sreng S, Anderson JM, Duong S, Nguon C, Chuor CM, Saunders D, Se Y, Lon C, Fukuda MM, Amenga-Etego L, Hodgson AV, Asoala V, Imwong M, Takala-Harrison S, Nosten F, Su XZ, Ringwald P, Ariey F, Dolecek C, Hien TT, Boni MF, Thai CQ, Amambua-Ngwa A, Conway DJ, Djimdé AA, Doumbo OK, Zongo I, Ouedraogo JB, Alcock D, Drury E, Auburn S, Koch O, Sanders M, Hubbart C, Maslen G, Ruano-Rubio V, Jyothi D, Miles A, O’Brien J, Gamble C, Oyola SO, Rayner JC, et al. 2013 Multiple populations of artemisinin-resistant *Plasmodium falciparum* in Cambodia. Nat Genet 45:648–655. doi:10.1038/ng.2624. 23624527 PMC3807790

[55] Ye R, Hu D, Zhang Y, Huang Y, Sun X, Wang J, Chen X, Zhou H, Zhang D, Mungthin M, Pan W 2016 Distinctive origin of artemisinin-resistant *Plasmodium falciparum* on the China-Myanmar border. Sci Rep 6:20100. doi:10.1038/srep20100. 26831371 PMC4735722

[56] Amaratunga C, Witkowski B, Dek D, Try V, Khim N, Miotto O, Ménard D, Fairhurst RM 2014 *Plasmodium falciparum* founder populations in western Cambodia have reduced artemisinin sensitivity *in* *vitro*. Antimicrob Agents Chemother 58:4935–4937. doi:10.1128/AAC.03055-14. 24867977 PMC4136061

[57] Fairhurst RM 2015 Understanding artemisinin-resistant malaria: what a difference a year makes. Curr Opin Infect Dis 28:417–425. doi:10.1097/QCO.0000000000000199. 26237549 PMC4612278

[58] Ekland EH, Schneider J, Fidock DA 2011 Identifying apicoplast-targeting antimalarials using high-throughput compatible approaches. FASEB J 25:3583–3593. doi:10.1096/fj.11-187401. 21746861 PMC3177575

