# Supplementary figures and images for: Plasmodium falciparum K13 Mutations Differentially Impact Ozonide Susceptibility and Parasite Fitness In Vitro
Source: mBio. 2017 Apr 11;8(2):e00172-17. doi: 10.1128/mBio.00172-17 (PMC5388803; doi:10.1128/mBio.00172-17)

## FIGURE S1

Artemisinin (ART)

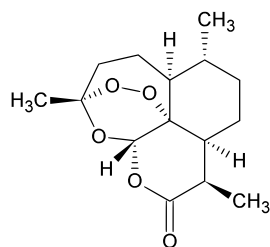

Dihydroartemisinin (DHA)

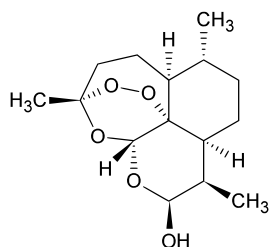

Arterolane (OZ277)

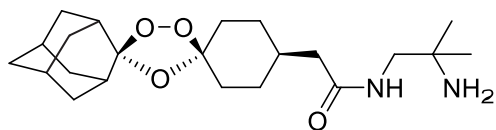

Artefenomel (OZ439)

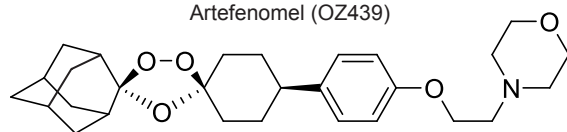

Supplement: FIG S1 [file mbo002173267sf1.pdf]

FIGURE S2

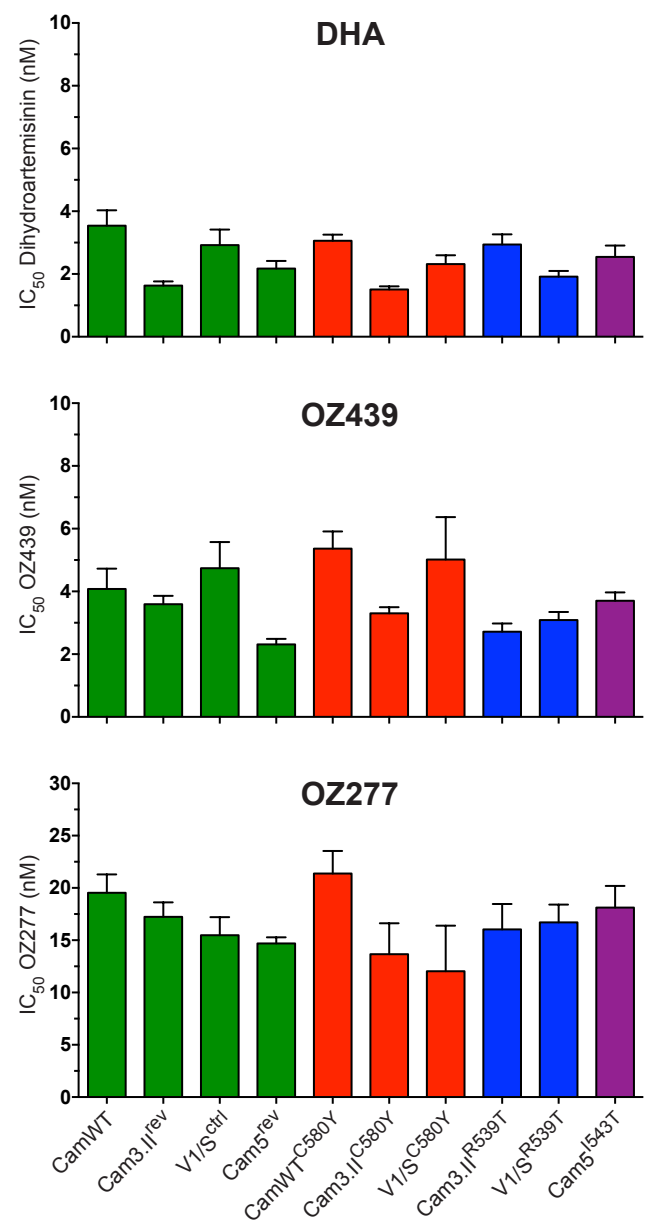

Supplement: FIG S2 [file mbo002173267sf2.pdf]

FIGURE S3

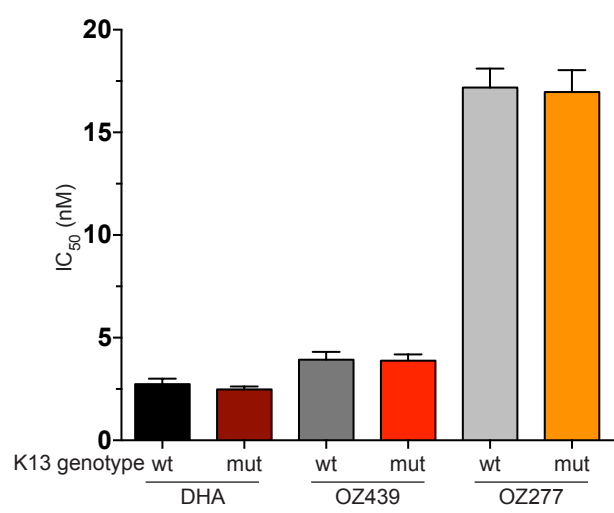

Supplement: FIG S3 [file mbo002173267sf3.pdf]

FIGURE S4

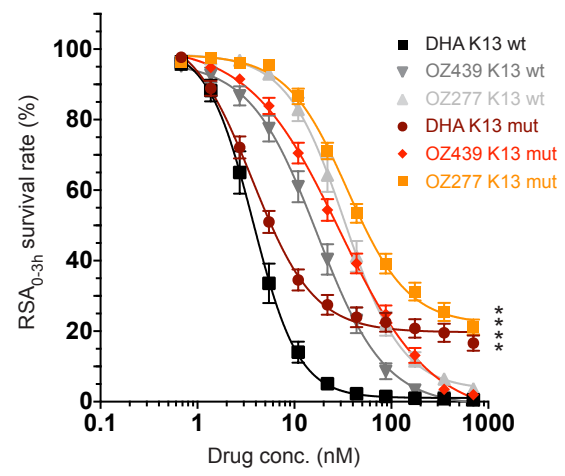

Supplement: FIG S4 [file mbo002173267sf4.pdf]
