# Supplementary material for: Plasmodium falciparum K13 Mutations Differentially Impact Ozonide Susceptibility and Parasite Fitness In Vitro
Source: mBio. 2017 Apr 11;8(2):e00172-17. doi: 10.1128/mBio.00172-17 (PMC5388803; doi:10.1128/mBio.00172-17)
Supplement: TABLE S1 [file mbo002173267st1.pdf]

TABLE S1. Geographic origin, native *K13* allele and drug-resistance genotypes of *Plasmodium falciparum* clinical isolates and reference lines.

Parasite	Original ID	Sanger ID	Provider	Geographic origin (year)	Gene-edited	$PCt_{1/2}$ (h)	KH group	K13-propeller	PMID #	*pfcrt*	*pfmdr1*	*pfmdr1* CN	*dhfr*	*dhps*
Cam3.II^{rev}		N/A			yes			synonomous mutation (wt)[a]	25502314	Dd2[b]	184F	1	Triple[c]	436A/540E
Cam3.II^{C580Y}		N/A			yes			C580Y (allele 2)	25502314	Dd2	184F	1	Triple	436A/540E
Cam3.II^{R539T}	RF 967	PH0306-C	R. Fairhurst	Pursat, W. Cambodia (2010)	no	6.0	3	R539T (allele 3)	22940027	Dd2	184F	1	Triple	436A/540E
V1/S^{ctrl}	V1/S	N/A	MR4	Vietnam (1976)	yes	N/A	N/A	synonomous mutation (wt)	12124623	Dd2	86Y	1	Quadruple	613T
V1/S^{C580Y}					yes	N/A	N/A	C580Y (allele 2)	(this report)	Dd2	86Y	1	Quadruple	613T
V1/S^{R539T}					yes	N/A	N/A	R539T (allele 3)	25502314	Dd2	86Y	1	Quadruple	613T
Cam5^{rev}		N/A			yes	N/A	N/A	synonomous mutation (wt)	25502314	Dd2	WT	1	Quadruple	436F/540E/613S
Cam5^{I543T}	IPC 4912	N/A	D. Ménard	Mondulkiri, E. Cambodia (2011)	yes	ND	ND	I543T (allele 5)	25502314	Dd2	WT	1	Quadruple	436F/540E/613S
CamWT	RF 915	PH0164-C	R. Fairhurst	Pursat, W. Cambodia (2010)	no	3.7	1	no mutation (wt)	22940027	Dd2	WT	1	Triple	436A/540E
CamWT^{C580Y}					yes	N/A	N/A	C580Y (allele 2)	25502314	Dd2	WT	1	Triple	436A/540E

[a] Synonymous mutations introduced into *k13*-specific zinc-finger nuclease binding site; wt: wild-type K13 amino acid sequence.

[b] Dd2: 74I/75D/76T/220S/271E/326S/356T/371I.

[c] Triple, 51I/59R/108N; Quadruple, 51I/59R/108N/164L.

$PCt_{1/2}$, parasite clearance half-life; PMID #, PubMed identification number; *pfmdr1* CN, *pfmdr1* copy number; MR4, Malaria Research and Reference Reagent Resource, Manassas, VA; N/A, not available; ND, not determined.
