# Supplementary material for: Plasmodium falciparum K13 Mutations Differentially Impact Ozonide Susceptibility and Parasite Fitness In Vitro
Source: mBio. 2017 Apr 11;8(2):e00172-17. doi: 10.1128/mBio.00172-17 (PMC5388803; doi:10.1128/mBio.00172-17)
Supplement: TABLE S2 [file mbo002173267st2.pdf]

TABLE S2. IC_{50} values for DHA, OZ439 and OZ277.

Parasite	DHA				OZ439				OZ277			
	IC_{50} Mean[a]	SEM	n	*p* value[b]	IC_{50} Mean	SEM	n	*p* value	IC_{50} Mean	SEM	n	*p* value
Cam3.II^{rev}	3.5	0.5	5		4.1	0.6	6		19.5	1.7	6	
Cam3.II^{C580Y}	3.1	0.2	6	0.3	5.4	0.5	4	0.2	21.4	2.2	5	0.5
Cam3.II^{R539T}	2.9	0.3	4	0.4	2.7	0.3	4	0.1	16.0	2.4	4	0.3
V1/S^{ctrl}	1.6	0.1	3		3.6	0.3	3		17.2	1.4	3	
V1/S^{C580Y}	1.5	0.1	3	0.5	3.3	0.2	3	0.4	13.7	3.0	4	0.4
V1/S^{R539T}	1.9	0.2	4	0.3	3.1	0.3	4	0.2	16.7	1.7	4	0.8
Cam5^{rev}	2.9	0.5	5		4.7	0.8	6		15.5	1.7	4	
Cam5^{I543T}	2.5	0.4	5	0.6	3.7	0.3	5	0.3	18.1	2.1	5	0.4
CamWT	2.2	0.2	3		2.3	0.2	3		14.7	0.6	3	
CamWT^{C580Y}	2.3	0.3	3	0.7	5.0	1.4	4	0.2	12.0	4.4	2	0.5

[a] Values listed are in nM. SEM, standard error of the mean. N = number of independent experiments.

[b] *p* values were calculated compared to the resepective isogenic reference lines Cam3.II^{rev}, V1/S^{ctrl}, Cam5^{rev} or CamWT.
