# Supplementary material for: Plasmodium falciparum K13 Mutations Differentially Impact Ozonide Susceptibility and Parasite Fitness In Vitro
Source: mBio. 2017 Apr 11;8(2):e00172-17. doi: 10.1128/mBio.00172-17 (PMC5388803; doi:10.1128/mBio.00172-17)
Supplement: TABLE S3 [file mbo002173267st3.pdf]

TABLE S3. Oligonucleotides used in this study.

Name	Nucleotide sequence (5'-3')[a]	Description	Lab name
p1	[Biotin-5] GTGGTGTTACGTCAAATGGTAGAA	I543T forward	p5389
p2	CCACCTCTACCCATGCTTTCATA	I543T reverse	p5390
p3	CCATCATATCCCCCA	I543T sequencing	p5391
p4	GGTAATAACTATGATTATAAGGC	R539T forward	p4668
p5	[Biotin-5]ACCTCTACCCATGCTTTCATACGA	R539T reverse	p4669
p6	GTGGTGTTACGTCAAATGGTA	R539T sequencing	p4670
p7	CTATTATACCGAATGTAGAAGC	C580Y forward	p4671
p8	[Biotin-5]AGGTAATTAAAAGCTGCTCCTGAA	C580Y reverse	p4672
p9	CCCCTAGATCATCAGCTATGT	C580Y sequencing	p4673
