# Supplementary material for: Plasmodium falciparum K13 Mutations Differentially Impact Ozonide Susceptibility and Parasite Fitness In Vitro
Source: mBio. 2017 Apr 11;8(2):e00172-17. doi: 10.1128/mBio.00172-17 (PMC5388803; doi:10.1128/mBio.00172-17)

*Plasmodium falciparum* K13 mutations differentially impact ozonide susceptibility and parasite fitness *in vitro*

Judith Straimer, Nina F. Gnädig, Barbara H. Stokes, Michelle Ehrenberger, Audrey A. Crane, David A. Fidock

Supplemental Statistics

Results of two-sample t-test with unequal variances, RSA_0-3h_ 21.88nM of drug, all K13 WT combined.

DHA vs OZ439

DHA vs OZ277

Results of two-sample t-test with unequal variances, RSA_0-3h_ 700nM DHA.

Cam3.II^rev^ vs Cam3.II^C580Y^

Cam3.II^rev^ vs Cam3.II^R539T^

V1S vs V1S^C580Y^

V1S vs V1S^R539T^

Cam5^rev^ vs Cam5^I543T^

CamWT vs CamWT^C580Y^

Results of two-sample t-test with unequal variances, RSA_0-3h_ 700nM OZ439.

Cam3.II^rev^ vs Cam3.II^C580Y^

Cam3.II^rev^ vs Cam3.II^R539T^

V1S vs V1S^C580Y^

V1S vs V1S^R539T^

Cam5^rev^ vs Cam5^I543T^

Cam5^rev^ vs Cam5^I543T^ (175nM)

CamWT vs CamWT^C580Y^

Results of two-sample t-test with unequal variances, RSA_0-3h_ 700nM OZ277.

Cam3.II^rev^ vs Cam3.II^C580Y^

Cam3.II^rev^ vs Cam3.II^R539T^

V1S vs V1S^C580Y^

V1S vs V1S^R539T^

Cam5^rev^ vs Cam5^I543T^

Cam5^rev^ vs Cam5^I543T^

CamWT vs CamWT^C580Y^

Results of two-sample t-test with unequal variances, RSA_0-3h_ 700nM DHA, all K13 WT vs. K13 MUT combined.

Results of two-sample t-test with unequal variances, RSA_0-3h_ 700nM OZ439, all K13 WT vs. K13 MUT combined.

Results of two-sample t-test with unequal variances, RSA_0-3h_ 700nM OZ277, all K13 WT vs. K13 MUT combined.

**Results of two-sample t-test with unequal variances, RSA_0-3h_ 21.88nM of drug, all K13 WT combined.**

DHA vs OZ439 (groups 1 vs. 2 respectively; p<0.0001; Figure 1)


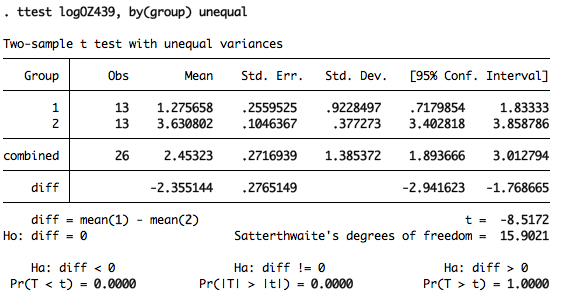


DHA vs OZ277 (groups 1 vs. 2 respectively; p<0.0001; Figure 1)


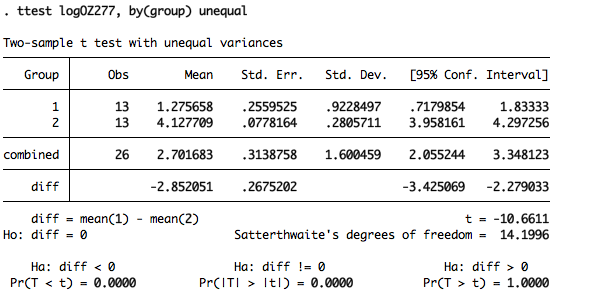


**Results of two-sample t-test with unequal variances, RSA_0-3h_ 700nM DHA.**


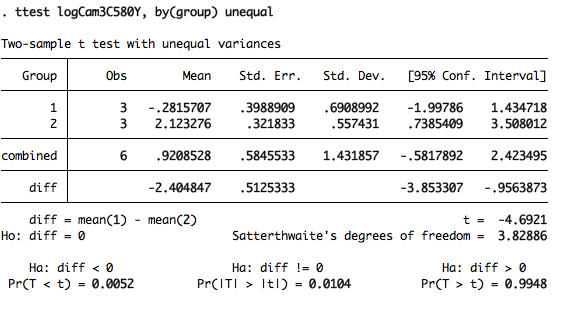
Cam3.II^rev^ vs Cam3.II^C580Y^ (groups 1 vs. 2 respectively; p<0.05; Figure 2)


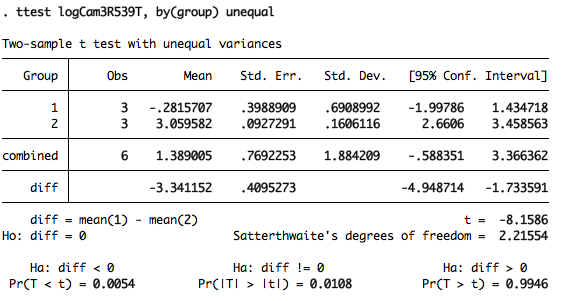
Cam3.II^rev^ vs Cam3.II^R539T^ (groups 1 vs. 2 respectively; p<0.05; Figure 2)

V1/S vs V1/S^C580Y^ (groups 1 vs. 2 respectively; p<0.05; Figure 2)


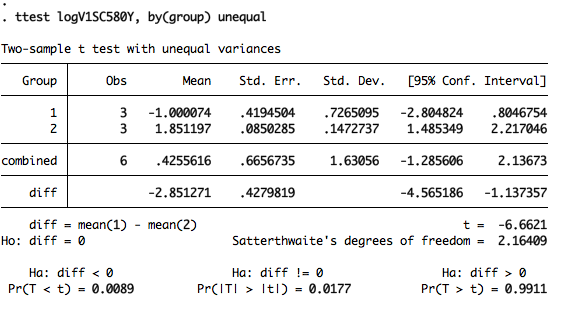


V1/S vs V1/S^R539T^ (groups 1 vs. 2 respectively; p<0.01; Figure 2)


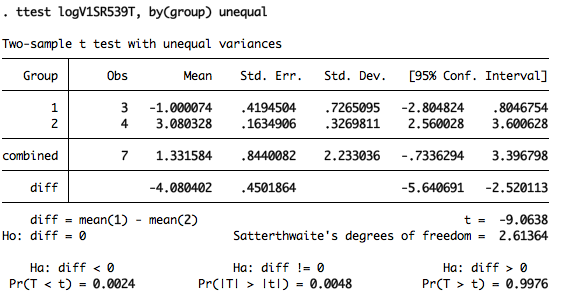


Cam5^rev^ vs Cam5^I543T^ (groups 1 vs. 2 respectively; p<0.0001; Figure 2)


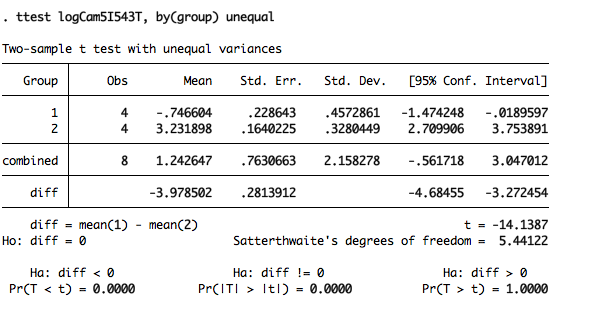


CamWT vs CamWT^C580Y^ (groups 1 vs. 2 respectively; p<0.001; Figure 2)


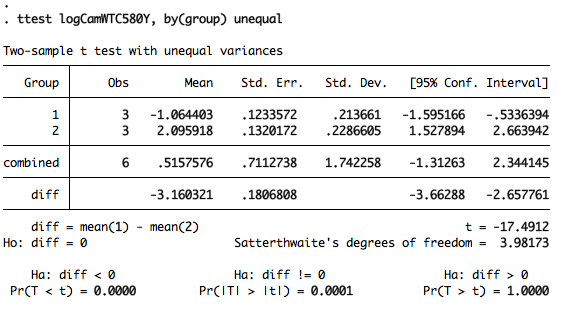


**Results of two-sample t-test with unequal variances, RSA_0-3h_ 700nM OZ439.**

Cam3.II^rev^ vs Cam3.II^C580Y^ (groups 1 vs. 2 respectively; p<1.0; Figure 2)


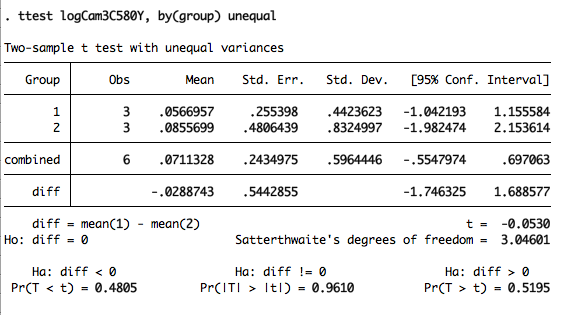


Cam3.II^rev^ vs Cam3.II^R539T^ (groups 1 vs. 2 respectively; p<1.0; Figure 2)


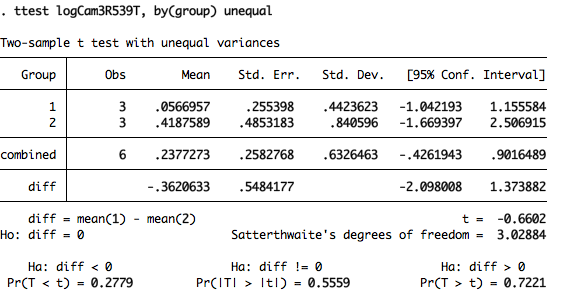


V1/S vs V1/S^C580Y^ (groups 1 vs. 2 respectively; p<1.0; Figure 2)


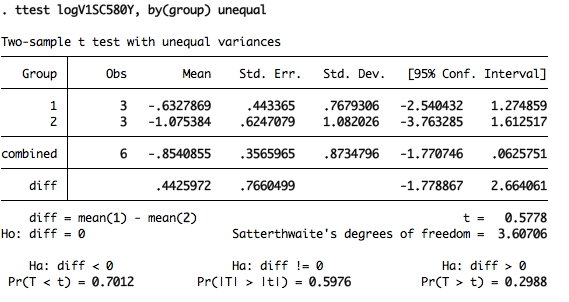


V1/S vs V1/S^R539T^ (groups 1 vs. 2 respectively; p<0.1; Figure 2)


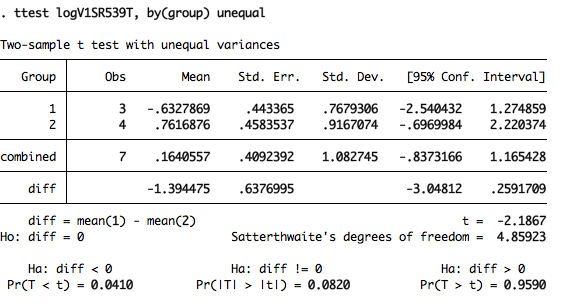


Cam5^rev^ vs Cam5^I543T^ (groups 1 vs. 2 respectively; p<0.05; Figure 2)


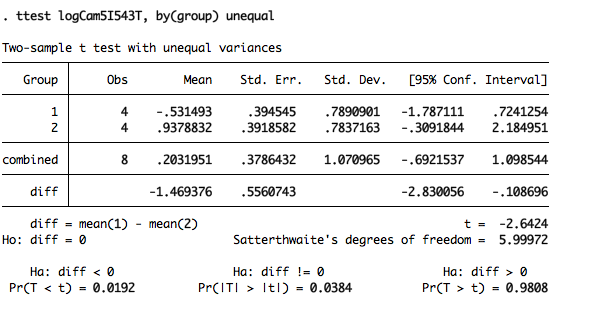


Cam5^rev^ vs Cam5^I534T^ (groups 1 vs. 2 respectively; p<0.001; Figure 2) 175nM


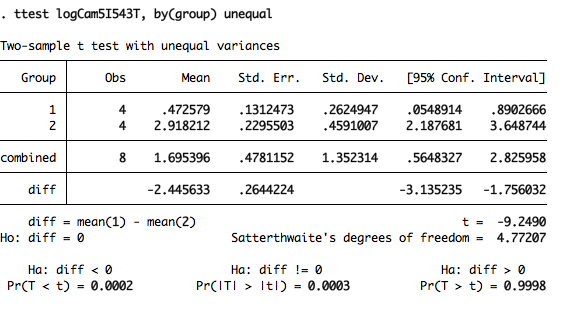


CamWT vs CamWT^C580Y^ (groups 1 vs. 2 respectively; p<0.1; Figure 2)

**
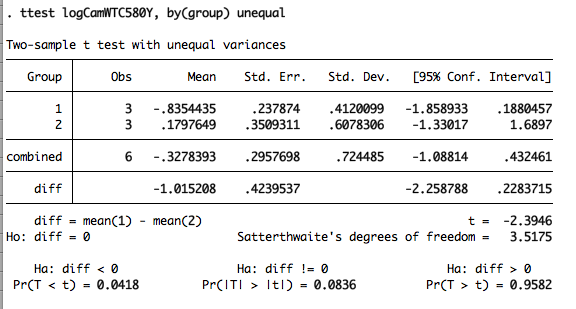
**

**Results of two-sample t-test with unequal variances, RSA_0-3h_ 700nM OZ277.**

Cam3.II^rev^ vs Cam3.II^C580Y^ (groups 1 vs. 2 respectively; p<0.1; Figure 2)


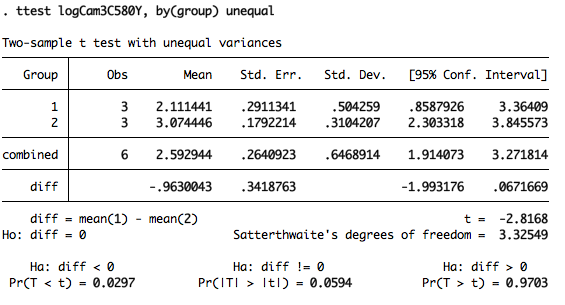


Cam3.II^rev^ vs Cam3.II^R539T^ (groups 1 vs. 2 respectively; p<0.05; Figure 2)


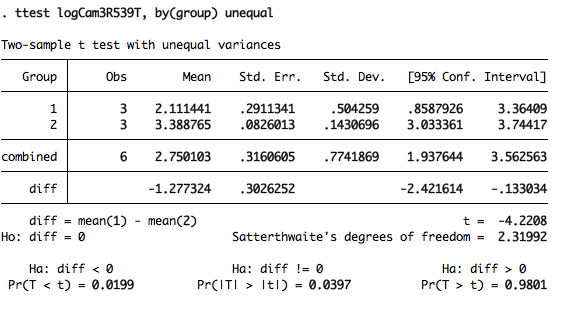


V1/S vs V1/S^C580Y^ (groups 1 vs. 2 respectively; p<0.01 Figure 2)


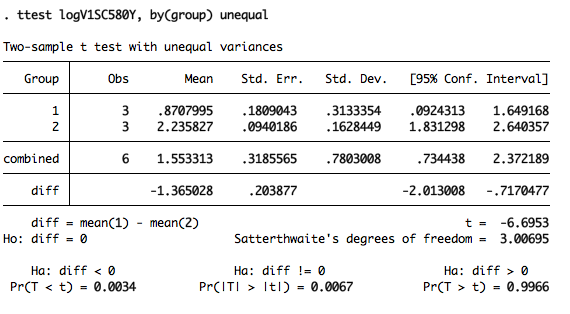


V1/S vs V1/S^R539T^ (groups 1 vs. 2 respectively; p<0.001; Figure 2)


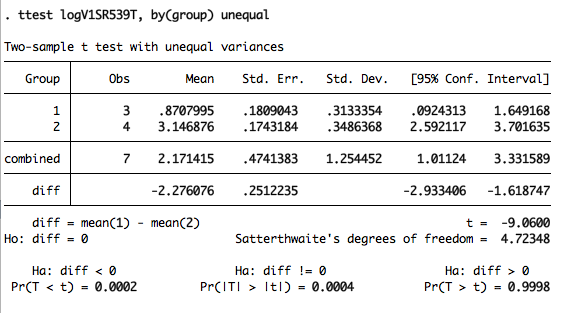


Cam5^rev^ vs Cam5^I543T^ (groups 1 vs. 2 respectively; p<0.001; Figure 2)


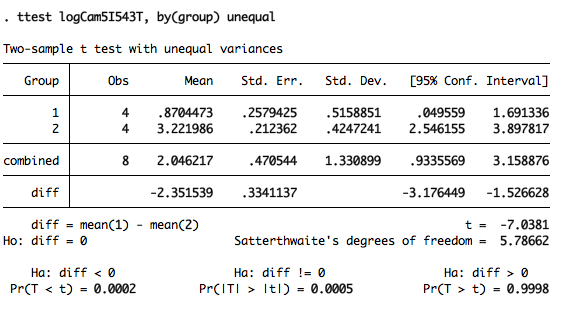


CamWT vs CamWT^C580Y^ (groups 1 vs. 2 respectively; p<0.05; Figure 2)


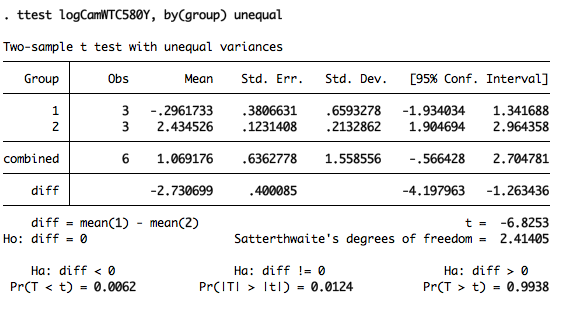


**Results of two-sample t-test with unequal variances, RSA_0-3h_ 700nM DHA, all K13 WT vs. K13 MUT combined.**

K13 WT vs K13 MUT (groups 1 vs. 2 respectively; p<0.0001; Figure 1)


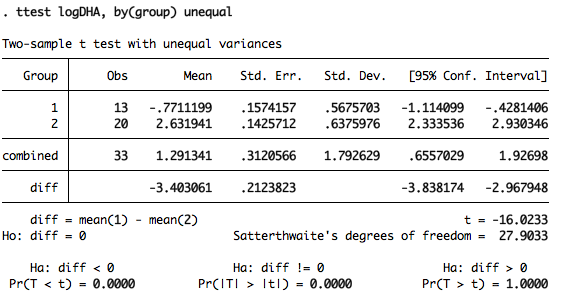


**Results of two-sample t-test with unequal variances, RSA_0-3h_ 700nM OZ439, all K13 WT vs. K13 MUT combined.**


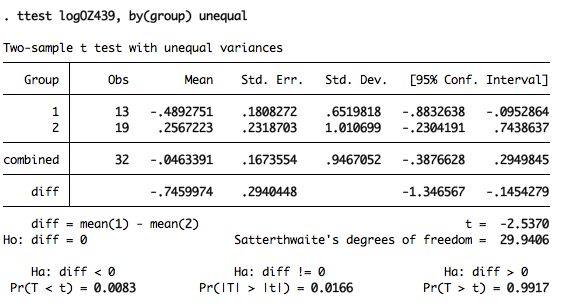
K13 WT vs K13 MUT (groups 1 vs. 2 respectively; p<0.0001; Figure 1)

**Results of two-sample t-test with unequal variances, RSA_0-3h_ 700nM OZ277, all K13 WT vs. K13 MUT combined.**

K13 WT vs K13 MUT (groups 1 vs. 2 respectively; p<0.0001; Figure 1)


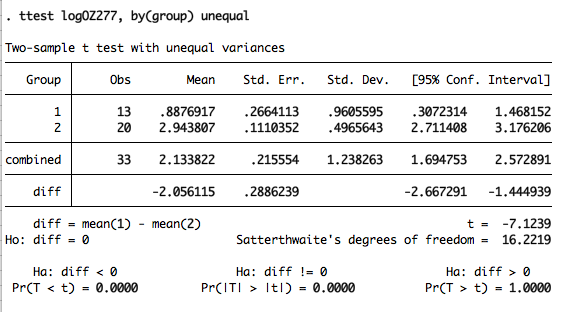

Supplement: TEXT S1 [file mbo002173267s1.docx]
